# Direct Estimation of the Mitochondrial DNA Mutation Rate in Drosophila melanogaster

**DOI:** 10.1371/journal.pbio.0060204

**Published:** 2008-08-19

**Authors:** Cathy Haag-Liautard, Nicole Coffey, David Houle, Michael Lynch, Brian Charlesworth, Peter D Keightley

**Affiliations:** 1 Institute of Evolutionary Biology, School of Biological Sciences, University of Edinburgh, Edinburgh, United Kingdom; 2 Department of Biology, Indiana University, Bloomington, Indiana, United States of America; 3 Department of Biological Science, Florida State University, Tallahassee, Florida, United States of America; University of Bath, United Kingdom

## Abstract

Mitochondrial DNA (mtDNA) variants are widely used in evolutionary genetics as markers for population history and to estimate divergence times among taxa. Inferences of species history are generally based on phylogenetic comparisons, which assume that molecular evolution is clock-like. Between-species comparisons have also been used to estimate the mutation rate, using sites that are thought to evolve neutrally. We directly estimated the mtDNA mutation rate by scanning the mitochondrial genome of Drosophila melanogaster lines that had undergone approximately 200 generations of spontaneous mutation accumulation (MA). We detected a total of 28 point mutations and eight insertion-deletion (indel) mutations, yielding an estimate for the single-nucleotide mutation rate of 6.2 × 10^−8^ per site per fly generation. Most mutations were heteroplasmic within a line, and their frequency distribution suggests that the effective number of mitochondrial genomes transmitted per female per generation is about 30. We observed repeated occurrences of some indel mutations, suggesting that indel mutational hotspots are common. Among the point mutations, there is a large excess of G→A mutations on the major strand (the sense strand for the majority of mitochondrial genes). These mutations tend to occur at nonsynonymous sites of protein-coding genes, and they are expected to be deleterious, so do not become fixed between species. The overall mtDNA mutation rate per base pair per fly generation in *Drosophila* is estimated to be about 10× higher than the nuclear mutation rate, but the mitochondrial major strand G→A mutation rate is about 70× higher than the nuclear rate. Silent sites are substantially more strongly biased towards A and T than nonsynonymous sites, consistent with the extreme mutation bias towards A+T. Strand-asymmetric mutation bias, coupled with selection to maintain specific nonsynonymous bases, therefore provides an explanation for the extreme base composition of the mitochondrial genome of *Drosophila*.

## Author Summary

Mitochondria are the energy-producing organelles of the cell, and they contain genetic information encoded on their own genome. Because rates of mutation for mitochondrial genomes are believed to be much higher than those in nuclear DNA, mitochondrial genetic differences between and within species are particularly useful in population genetics, for example, as markers of population movements. We have directly estimated the mutation rate in the mitochondrial genome of the fruit fly Drosophila melanogaster in lines that had been allowed to randomly accumulate mutations in the virtual absence of effective natural selection. We scanned for new mutations by comparing the DNA of different lines by a sensitive mutation detection technique. We show that the mitochondrial mutation rate is about ten times higher than the nuclear DNA mutation rate. Strikingly, however, almost all of the single–base pair mutations that we detected change G to A at an amino acid site of a protein-coding gene. The explanation for this effect seems to be that natural selection maintains the nucleotide G at amino acid sites, whereas most silent sites are under weaker selection and have previously mutated to A or T. The mutation rate for G to A changes is 70 times higher than the nuclear DNA mutation rate. This extreme mutation bias maintains the high A+T content of the *Drosophila* mitochondrial genome.

## Abstract 3

We show that the mitochondrial DNA mutation rate in *D. melanogaster* is 10X higher than the nuclear mutation rate, and that major-strand G-->A hypermutablity explains the extremely biased base composition of the mitochondrial genome.

## Introduction

Mitochondrial genetic variation between populations and species is widely used in dating evolutionary events and population movements [1]. These studies exploit several features of the mitochondrial genome, including its simple organization, lack of recombination, maternal mode of inheritance in many species, and, in animals, a high mutation rate relative to the nuclear genome [2,3].

In metazoans, the high mutation rate of the mitochondrial genome may be caused by a low efficiency of DNA repair pathways or by a more mutagenic intracellular environment. This results, for example, in a mean mitochondrial DNA (mtDNA) divergence at synonymous sites between species of vertebrates that is 5–50 times higher than in the nuclear genome [3]. In humans, the mitochondrial mutation rate may be even higher than interspecific divergences suggest, because human pedigree studies suggest a 10-fold higher rate than divergence-based estimates, based on the appearance of de novo mtDNA variants [4]. This discrepancy suggests that many mtDNA mutations may be subject to weak selection, which can occur either at the level of the population of individual females or within the germ line [5]. The difficulty in estimating the mutation rate has hampered theoretical understanding of the maintenance of the nonrecombining mitochondrial genome in the face of a continual flux of deleterious mutations, which could lead to genetic degradation via Muller's Ratchet [3,6]. It also has led to controversy concerning the use of divergence between mtDNA variants as a proxy for the mutation rate, with consequences for the dating of evolutionary events [4]. On a more practical level, mitochondrial defects are an important cause of human genetic disease [7]. For example, more than 100 different mtDNA point mutations are associated with disease, and these display a wide range of phenotypes [8].

Despite its small genome size, natural variation in *Drosophila* mtDNA genotypes has been shown to affect fitness in the laboratory [9]. In contrast to mammals, however, estimates in *Drosophila* of the silent-site divergence for the nuclear and mitochondrial genomes are quite similar to one another [10,11]. The D. melanogaster mitochondrial genome, in common with that of many other insect taxa [12], has a biased base composition (82% A+T overall), particularly at 4-fold degenerate synonymous sites (94% A+T). Strong mutation bias can make it difficult to accurately estimate substitution rates from interspecific DNA sequence comparisons of silent sites. Further uncertainty concerning the mutation rate is associated with the potential for purifying selection to reduce the substitution rate. Additionally, mutation rate inference based on between-species substitution rates relies on estimates of the generation time and between-species divergence time, both of which may be difficult to determine with confidence.

Here, we measure rates and properties of new mutations for the D. melanogaster mitochondrial genome in a setting that is largely free from selection at the level of individual flies. We use two strategies—direct sequencing and denaturing high performance liquid chromatography (DHPLC)—to scan the mitochondrial genome of mutation accumulation (MA) lines in which the effectiveness of selection at the population level has been reduced by close inbreeding (mostly full-sib mating) for many tens of generations. Most of the mutations we find are heteroplasmic, which we characterise by pyrosequencing. Our results shed light on the origins of the extremely biased base composition of the *Drosophila* mitochondrial genome.

## Results

### Scanning the Mitochondrial Genome for New Mutations by Direct Sequencing and DHPLC

We scanned D. melanogaster mitochondrial genomes of three sets of MA lines [Madrid, Florida-33 (F-33), and Florida-39 (F-39)] by DHPLC and of two sets (F-33 and F-39) by direct sequencing. The two mutation detection methods were run independently of one other. The mutations detected by DHPLC were subsequently confirmed and characterised by direct Sanger sequencing of the affected line (sequence traces of mutants and wild types are shown in Protocol S1). The frequencies of all mutations within a line were estimated by pyrosequencing or from the heights of sequence traces. Numbers of MA lines of each genotype and bases scanned are shown in Table 1. There was considerable overlap between the bases of the Florida lines scanned by the two methods, although somewhat more were scanned by direct sequencing than by DHPLC (Table 1). Over the whole experiment, a total of 42 variants were detected (Tables 2 and 3). A (TA)_6_→(TA)_7_ variant at position 5,970 (Table 3) was found segregating at different frequencies in five of the 32 F-33 MA lines (but not in lines of any other ancestral genotype). It therefore seems likely that these variants were present in the expansion phase of the progenitor inbred line from which the F-33 MA lines were derived, and we did not include these variants as genuine de novo mutations in subsequent analyses. An indel variant at position 2,877, segregating in two Madrid lines, was assumed to be a result of cross-contamination within the Madrid MA experiment and was counted as a single event. At positions 8,192 and 13,136, we detected indel variants that were segregating in different pairs of F-39 and Madrid lines. The affected line pairs differed for at least one other site in the same or a different amplicon, and the genotypes of these sites were consistent with other Madrid or Florida lines. The pairs of variants at sites 8,192 and 13,136 were therefore assumed to be genuine independent mutation events. The majority of the 36 events that we considered to be genuine de novo mutations involved a change of a single nucleotide (28 events), whereas the remainder (eight events) were indels that invariably involved changes in the repeat numbers of homopolymer or microsatellite-like sequences (Table 3).

**Table 1:**
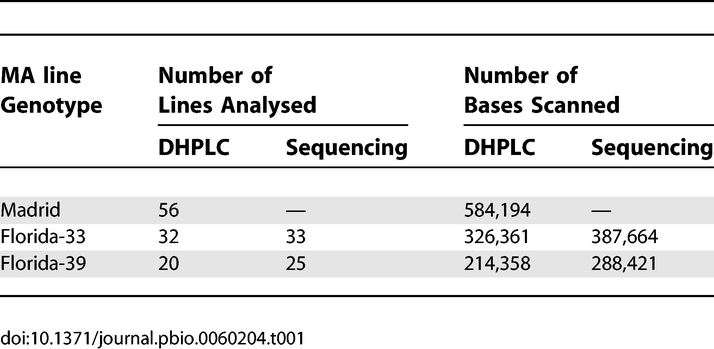
Number of MA Lines Analysed and Numbers of Bases Scanned by DHPLC and Direct Sequencing

**Table 2:**
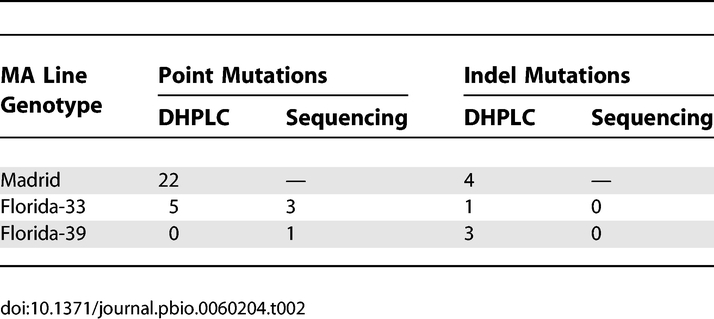
Numbers of Unique Mutation Events Detected

**Table 3:**
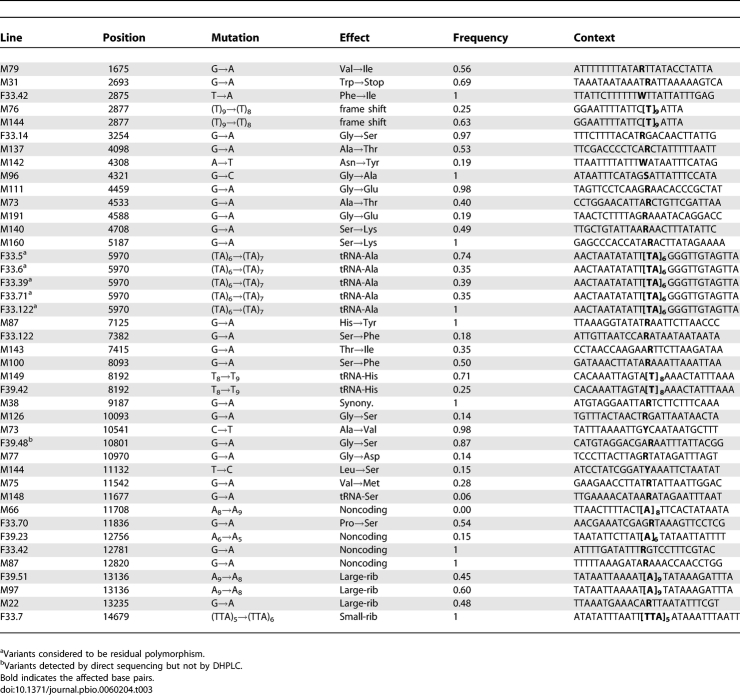
Details of the Variants Detected, Shown as Changes on the Major Strand

**Figure 1:**
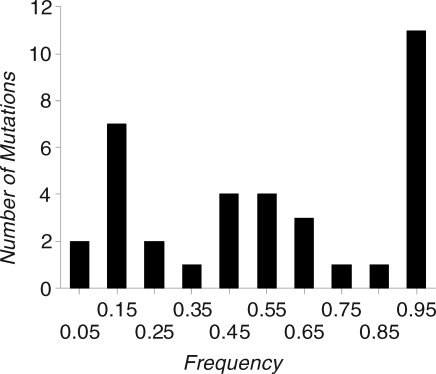
Distribution of Frequencies of Mutations The distribution of estimated frequencies of mutations that were detected by DHPLC, including one mutation that was detected by sequencing and not by DHPLC.

Under a neutral or purifying selection model at equilibrium, the distribution of mutation frequencies at segregating sites is expected to be L-shaped, with a peak close to zero [13]. However, the left peak of the observed distribution of the estimated frequencies of mutations (Figure 1) is between 0.1 and 0.2, and there are few mutations with frequencies in the range 0–0.1. Presumably, the observed distribution was affected by a failure to detect mutations segregating at low frequencies, whereas high-frequency mutations could be detected because the ancestral state is known. We investigate the effect of the tendency to miss low frequency mutations on our estimates below.

### Comparison between the Mutation-Detection Methods

The mitochondrial genome of the Florida MA lines was independently scanned by DHPLC and by direct sequencing, so this gives an opportunity to compare the efficiencies of the two methods for mutation detection. The results (Table 2 and Tables S1 and S2) suggest that DHPLC was superior to sequencing in the present experiment, because five mutations detected by DHPLC were not detected by sequencing of the corresponding regions. The mutations detected by DHPLC are unlikely to be false positives, because they were confirmed by direct sequencing of the affected lines and by pyrosequencing using independent PCRs each time. Two of the mutations detected by DHPLC but not by sequencing were segregating at low frequencies; this will often generate differences in sequence traces that are difficult to discern. A single mutation was detected by sequencing, but not by DHPLC of the corresponding genomic segment. A low rate of failure to detect mutations by DHPLC has been noted previously [14–16]. We use the DHPLC results in subsequent analysis, augmented by the one mutation that was only detected by direct sequencing.

### Estimates of mtDNA Mutation Rate and Effective Population Size

Few mutations were detected in the Florida MA lines, so we pooled F-33 and F-39 data (which were derived from a common base population) in the analysis reported below. We estimated the overall mutation rate per site per fly generation (μ) by a simple approximation of the ratio of the sum of the estimated frequencies of the mutations within lines to the total number of bases scanned (Equation 2). The new mutations that arise in a line each generation will drift to some frequency distribution through time that is defined by the intracellular effective population size, *N*_e_ [17]. Given the observed frequency distribution of mutations segregating within lines after *t* generations of MA, it should then be possible to obtain joint estimates of *N*_e_ and the mutation rate, assuming neutrality. We developed such a method, based on maximum likelihood (ML), which assumes that the frequencies are known with error, where the error distribution is a truncated normal distribution with a variance of the untruncated distribution *V*_E_. The results of applying the approximate and ML methods are highly consistent in all cases (Table 4). The overall mutation rate estimates are somewhat higher in the Madrid than in the Florida lines, but a model in which the Florida and Madrid data are analysed together with the same mutation rate (but different *N*_e_ and *V*_E_) fits only marginally worse by ML than a model with different mutation rates (i.e., differences in log *L* are equal to 0.9 and 0.0 for point mutations and for all types of mutations, respectively). Mean ML mutation rate estimates are 6.2 × 10^−8^ and 1.6 × 10^−8^ per site per fly generation for single base and indel events, respectively. These rates are about one order of magnitude higher than the estimates for the nuclear genome of the same MA lines [16]. Given our estimates of the mutation rate, we would not expect to detect mutational hotspots in our data, unless the hotspots were extremely strong.

**Table 4:**
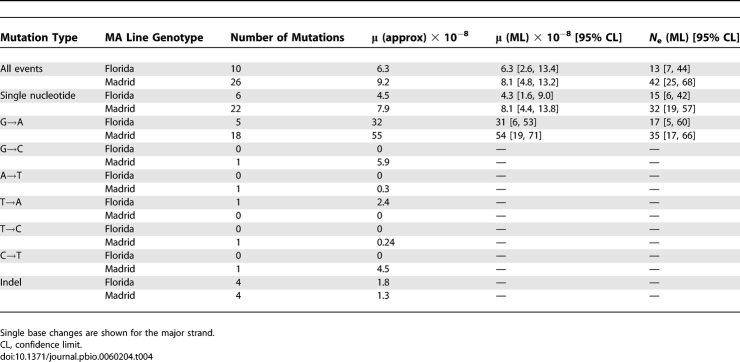
Estimates of per Site per Generation Mutation Rates and Effective Population Size per Fly Generation for the Mitochondrial Genome

Our results clearly demonstrate substantial heterogeneity in the mutation rate among the four nucleotides. The most striking example is the high frequency of G→A mutations on the major strand, which is the sense strand for the majority of mitochondrial genes (Table 3). Among the single-nucleotide mutations, there is a strong transition:transversion bias of 25:3. In spite of the A+T richness of the genome (82% for the Genbank U37541 reference sequence), there is a pronounced G/C→A/T:A/T→G/C bias of 24:1 among the transitions. The majority of sites that were scanned are in protein-coding DNA. Of the 24 single-nucleotide mutations detected in coding DNA, all but one were nonsynonymous (Table 3). This is explained by the mutational bias in favour of A+T and the extreme A+T richness of synonymous sites in the mitochondrial genome (i.e., 94% of codons end in T or A; [18]). There were 23 major-strand G→A mutations, compared to only five major-strand single-nucleotide mutations of all other types (χ^2^ = 1 degree of freedom (d.f.) = 11.6; *p* < 0.001). The mean ML major-strand G→A mutation rate estimate is 4.2 × 10^−7^ (Table 4), compared to a mean estimate of 1.2 × 10^−8^ for all other single-nucleotide changes. Furthermore, in spite of the high A+T content of the genome, 24 events would increase A+T content, whereas only a single event would decrease A+T content. We cannot, therefore, obtain a precise estimate of the predicted equilibrium A+T content, although it is very close to 1. This implies that selection must maintain G and C bases in coding sequences in the D. melanogaster mitochondrial genome, possibly to enhance the efficiency and accuracy of translation [19].

### Effective Population Size

The ML procedure estimates the effective number of maternal mitochondria transmitted to progeny. Estimates of *N*_e_ (Table 4) are in the range 13–42, which implies that drift within individuals will be important in determining the fates of new mutations, unless they have fitness effects in excess of several percent within an individual. Confidence intervals for effective size overlap between Madrid and Florida. Simulation results (see below) suggest that *N*_e_ may be underestimated if mutations of low frequency were not detected, which is undoubtedly the case.

### Simulations

**Figure 2:**
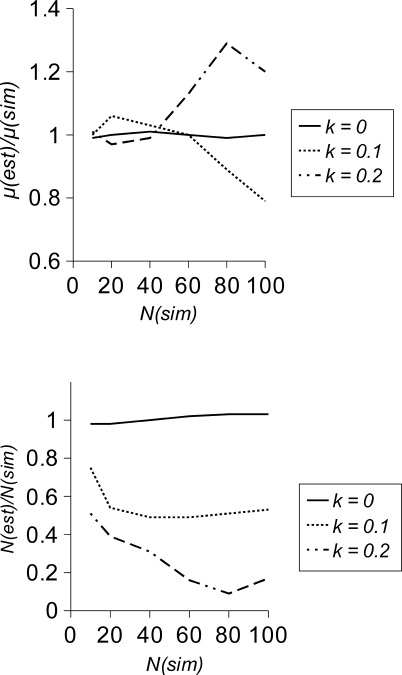
Simulation Results Simulation results for the ratio of mean estimate/simulated value of μ (upper panel) and *N* (lower panel) for three values of *k* (the frequency below which mutations are rejected) as a function of the population size simulated, *N*(*sim*). Other parameters were μ = 10^−6^, *V*_E_ = 0.001, *t* = 200, and 10^5^ sites. Each point is the mean of 100 replicates.

We investigated the performance of the ML inference method using simulations, in which mutations were assumed to be undetectable when their frequency within a line was below a value *k*. The results (Figure 2) suggest that if all mutations are detected (*k* = 0), estimates of μ and *N*_e_ are essentially unbiased. However, if mutations with low frequencies are missed (i.e., *k* = 0.1 or 0.2 in the cases simulated), estimates of μ can become biased (i.e., upward or downward by ∼30% in the cases simulated). If *N*_e_ ≪ *t*, where *t* is the number of generations of MA, then most of the information to estimate μ comes from fixed mutations, so excluding segregating mutations produces little bias. Bias becomes increasingly important as *N*_e_ approaches *t* (corresponding to the right hand side of the figures). Surprisingly, the bias affecting estimates of μ is in opposite directions for *k* = 0.1 and *k* = 0.2. For small values of *k*, μ is underestimated because part of the distribution of mutation frequencies is missing. However, for higher values of *k*, μ can be overestimated because *V*_E_ can then become numerically unstable and be dramatically overestimated, which leads to a large underestimation of *N*_e_. In analysing the real data, we penalise implausible values of *V*_E_, so this problem of numerical instability did not occur. If the frequency distribution is truncated, estimates of *N*_e_ are consistently downwardly biased, particularly if the simulated *N*_e_ is large.

## Discussion

The D. melanogaster mitochondrial genome has an extremely biased base composition, containing 82% A+T. At both 2-fold and 4-fold degenerate sites, base composition is even more extreme at 94% A+T, compared to 66% A+T at 0-fold sites. On the major strand (the sense strand for nine of the 13 protein-coding genes), 4-fold degenerate sites also have conspicuous excesses of A (56%) and C (4.0%) over T (38%) and G (1.9%), respectively, and 2-fold degenerate sites have an excess of C (4.5%) over G (1.1%). These genomic and strand-specific compositional biases can be understood in the light of several features of our results. First, 24/28 of our single-base mutations are G/C→A/T (Table 3). This is consistent with the A+T bias of the genome as a whole, and especially with the bias at synonymous sites, whose composition is presumably strongly affected by mutation. Second, all but one of the 25 mutations in protein-coding sequences are nonsynonymous (Table 3). This can be attributed to the 5-fold higher G+C content at amino acid sites compared to synonymous sites, coupled with the G→A mutation bias. The higher G+C content at amino acid sites implies that selection maintains amino acids that are encoded by G or C at the first and second codon positions. Third, the high proportion of G→A mutations on the major strand is consistent with the higher proportion of A and C than T and G on the major strand at synonymous sites, particularly at 4-fold degenerate sites. For example, the Cyt b gene, which is encoded on the major strand, shows a substantially lower frequency of G-ending codons than the minor strand-encoded *ND5* gene [20]. Furthermore, there are 23 major-strand G→A mutations, compared to only a single major-strand C→T mutation, which is consistent with the higher proportion of C than G at 4-fold and 2-fold degenerate sites. Asymmetric mutation is believed to generate strand-specific compositional asymmetry as a result of the mechanism of replication of the mitochondrial genome, in which the minor strand remains single stranded for longer than the major strand, and is thereby more prone to mutations [21,22].

The presence of base-specific and strand-specific mutational biases in the mitochondrial genome has previously been inferred from within- and between-species sequence comparisons [20,23,24]. Ballard [24] polarised substitutions among several members of the D. melanogaster subgroup using parsimony, and reports a greater number of major strand A→G substitutions than G→A substitutions at synonymous sites, whereas we see no A→G mutations, and an excess of G→A mutations at nonsynonymous sites. This difference presumably reflects the strong compositional bias at synonymous sites (which have very few G bases that can mutate to A). It may also be influenced by the tendency for parsimony to misassign changes towards the rarer base, if there is biased base composition, as is the case here [25,26]. Ballard [24] also reports a major-strand excess of synonymous C→T substitutions compared to G→A substitutions, whereas we saw only a single, nonsynonymous C→T mutation (Table 3). This may partly reflect the lower frequency of major-strand synonymous G sites compared to C sites.

Synonymous-site divergence between D. simulans and D. melanogaster for the mitochondrial and nuclear genomes has been estimated to be close to 0.12 for both [10,11]. However, our estimates of the single-nucleotide mutation rate for the mitochondrial and nuclear genomes differ more than 10-fold, i.e., 6.2 × 10^−8^ (this study) and 5.8 × 10^−9^ [16], respectively. The apparent discrepancy between the relative genome-wide mutation rates and relative synonymous site divergences can be at least partly explained by the difference in base composition between the mitochondrial genome as a whole and its synonymous sites. Mitochondrial synonymous sites are extremely A+T-rich and so are expected to mutate at a lower frequency than the mitochondrial genome as a whole, which is consistent with the low frequency of synonymous mutations that we observed (Table 3). Our high mitochondrial mutation rate estimate largely comes from mutations at nonsynonymous major-strand G sites; these are subject to strong purifying selection in nature, and this contribute little to between-species divergence.

In humans and other species, pedigree analysis has suggested a substantially higher mitochondrial mutation rate than the rate indirectly inferred from between-species phylogenetic comparisons [4,27]. The human mitochondrial genome as a whole and the control region are much less biased in their composition than D. melanogaster (i.e., 56% and 53% A+T, respectively), suggesting that mutational biases are not as strong in the human mitochondrion as in *D. melanogaster.* This is consistent with the broad spectrum of new variants seen in human pedigree studies [4]. Mutation bias is not therefore a strong candidate to explain the difference between pedigree and phylogenetic mtDNA mutation rate estimates. Howell et al. [4] and Ho et al. [27] discuss several other possible explanations for this discrepancy, including non-neutral evolution [28].

A MA line based direct estimate of the mtDNA mutation rate was carried out by Denver et al. [29] in Caenorhabditis elegans strain N2, using a direct sequencing approach. The estimate for the single-nucleotide mutation rate is also substantially higher than the corresponding estimate for the nuclear genome [30], and is about 1.5× higher than our estimate for the D. melanogaster mitochondrial genome. The C. elegans mitochondrial genome is A+T-rich (76%), although not as A+T-rich as the D. melanogaster genome (82%). However, of the 16 single-nucleotide mutations detected by Denver et al. [29] only four would increase A+T content, whereas the corresponding figure for our study is 24/28. In this respect, therefore, the outcomes of the two studies were quite different, suggesting that selection and mutation may be of different relative strengths in the two species. Only one heteroplasmic mutation was detected by Denver et al. [29] , although such events may be missed by direct sequencing, as in the present study. Alternatively, the low frequency of hetereroplasmic mutations suggests a lower effective population size for the C. elegans mitochondrial genome. Recent analysis of spontaneously arising mitochondrial mutations in MA lines of yeast (Saccharomyces cerevisiae) also supports the contention that mitochondrial mutational spectra differ substantially across species [31]. In this study, every mitochondrial base-substitutional mutation detected was in the direction of A/T→G/C, despite the strong (∼84%) A/T bias in the yeast mitochondrial genome.

Our estimate of the effective population size for the mitochondrial genome per fly generation can be compared with that of Solignac et al. [32], who studied changes in the variance of the frequency of two mtDNA alleles in the offspring of a heteroplasmic D. mauritiana female. Solignac et al. [32] estimated that the effective number of mtDNA genomes per cell generation lies between 545 and 700. Assuming that the effective population size per fly generation is inversely proportional to the cumulative drift from 7–9 germ cell divisions per individual generation [32], this gives a range for the effective number of mtDNA genomes per germ cell generation of 60–100, which is 2–3 times higher than our mean estimate of about 30 (Table 4). This difference is probably at least partly explained by a failure to detect low frequency variants in our experiment. However, Drost and Lee [33] suggest that the number of germline cell divisions is ∼36, which implies an *N*_e_ estimate close to ours.

Under the assumption that amino acid and frameshift mutations are unconditionally deleterious, an estimate for the deleterious mutation rate per mitochondrial genome (*U*_mt_) can be obtained from Σ *d_i_*/(*t* × *lines* × *p*), where *d_i_* is the observed frequency of a mutation within the MA lines (from Table 3), the summation is over amino acid-altering mutations, *lines* is the number of MA lines, and *p* is the proportion of amino acid sites in the mitochondrial genome that we scanned for mutations (0.64). This gives a mean estimate for the Madrid and Florida MA lines of *U*_mt_ = 8 × 10^−4^. However, noncoding mutations are not included in this calculation, and natural selection within individuals would cause both μ and *U*_mt_ to be underestimated. This is more likely to be an issue for mitochondrial mutations than for nuclear mutations because of the moderately high effective number of mitochondrial genomes within individual females.

Our analysis was limited for several reasons. First, most of the mutations we detected were segregating (i.e., heteroplasmic), and the shape of the frequency distribution of these suggests that we must have failed to detect many low-frequency mutations. The exact extent of this underestimation cannot be determined, because we do not know the contribution of the missing part of the distribution. Second, most of the mutations are nonsynonymous, which are expected to be subject to negative selection in nature, and it is likely that at least some of these are kept at low frequencies in the MA experiments. Such negative selection on mtDNA variants has recently been demonstrated in mice [34,35]. Finally, parts of the *Drosophila* mitochondrial genome are so A+T-rich that we were unable to amplify and scan these regions for new mutations. The single-nucleotide mutation rate to single nucleotide events is expected to be lower in these regions than in the relatively G+C-rich regions that we were able to scan. The recent emergence of massively parallel new sequencing technologies [36,37] that, in principle, allow the sequencing of regions of arbitrary base composition at a very high depth of coverage may be better suited than the technologies used in the present study. They should allow more accurate estimation of both the frequency distribution of segregating mutations at coding and silent sites and the mutation rate.

## Materials and Methods

### Mutation accumulation lines.

The mutation rate for the D. melanogaster mitochondrial genome was directly estimated in sets of MA lines of three genotypes—Madrid [38], Florida-33, and Florida-39 [39]—described in [16]. The Madrid MA line inbred progenitor was created using balancer chromosomes [40]. The Florida line progenitors were generated by 40 generations of full-sib mating [39]. The Madrid and Florida MA lines then experienced an average of 262 and 187 generations of MA, respectively. In the present analyses, four Madrid genotype lines were excluded, because the previous study on nuclear DNA had suggested breeding contamination within the experiment [16]. The two Florida MA line genotypes were derived from the same base population. Little DNA was available for several of the Madrid lines, so DNA samples from all the Madrid MA lines were amplified using a Repli-g whole-genome amplification kit (Qiagen) prior to analysis. Whole-genome amplified DNA was used for all of the DHPLC analyses, but whenever possible, sequencing was done on the original DNA samples. Whole-genome amplification used high-fidelity polymerase and a large excess of template, and does not generate mutations at a rate that would be detectable in our experiment [41]. In all cases, mutations detected by DHPLC (using whole-genome amplified DNA) were also detected by sequencing, whenever unamplified genomic DNA was used as template.

### Mutation detection overview.

We used two approaches to detect new mitochondrial mutations. (1) For the Florida lines, we directly sequenced amplicons of *∼*940 bp, and compared the sequence traces among lines for differences that would indicate the presence of a new mutation [29]. (2) For the Madrid and Florida lines, we used DHPLC to scan PCR-amplified segments of ∼700 bp for new mutations, as described by Haag-Liautard et al. [16]. Mutation detection was carried out independently by DHPLC and direct sequencing. Mutations detected by DHPLC were confirmed and characterised by direct sequencing of both strands of the affected amplicon. Details of the regions screened and the primers used in each technique can be found in the supplementary material (Tables S1 and S3).

### Mutation detection by direct sequencing.

For each MA line, 16 amplicons were amplified by PCR, and sequenced using the primers listed in Table S3, whose sequences were kindly provided by D. Rand and D. Abt. The amplicons are partially overlapping, and cover 12,199 bp (63% of the total mtDNA molecule). None of the amplicons overlap the noncoding region containing the site of initiation of replication, which is also known as the AT-region. PCR products were on average 940 bp long (range 605-1159 bp). For each amplicon, 100 ng of template was PCR-amplified using Eppendorf MasterMix Taq, and the length and quality of products verified on 1.5% agarose gels. PCR products were sequenced in both directions, but forward and reverse sequences overlapped only slightly, so can therefore be regarded as single stranded. Sequences were compared using CodonCode Aligner (version 1.5) software. Failed and poor quality sequences were excluded from the analyses.

### Mutation detection by DHPLC.

Fifteen regions of the mtDNA were amplified by PCR (see Tables S1 and S3) under the conditions described by Haag-Liautard et al. [16], except that for some amplicons giving unclear DHPLC profiles, we used Optimase Polymerase (Transgenomic) following the conditions recommended by the manufacturer. Amplicons, each 300–750 bp (average 678 bp), covered 11,428 bp (59%) of the mitochondrial DNA molecule (Table S1). We scanned the PCR-amplified segments for mutations by DHPLC using a Transgenomic Wave 3500a instrument following the methods described by Haag-Liautard et al. [16]. DHPLC was carried out using mixtures of four lines. Whenever comparison of the DHPLC traces among groups of MA lines suggested the presence of a mutation, the affected line was identified by a further round of DHPLC using all the pairwise combinations of the four lines, and these MA lines were then sequenced on both strands from new PCR products. In cases where the mutation appeared to be at a low frequency, the sequencing was repeated using an independent PCR as template to confirm its presence and its nature. In our previous study of the mutation rate in the nuclear genome, we used synthetic positive controls to measure the rate at which DHPLC fails to detect genuine fixed mutations, which was of the order of 2% [16].

### Estimation of mutation frequencies.

We used pyrosequencing [42] to estimate the frequencies of mutations, the majority of which appeared to be segregating within a line. PCR primers around a mutation and pyrosequencing primers were designed using the Biotage assays software. The sequence at and around the mutation was analysed with a Biotage pyrosequencing instrument, and the frequency of each allele at the defined mutation point estimated using the software provided by the manufacturer.

In some cases, it was difficult to estimate the mutant frequency by pyrosequencing (e.g., when a homopolymer was adjacent to the mutation), so we estimated the frequency based on a peak height comparison of the DNA sequence traces. We attempted to take into account the effect of the preceding base on peak height using the following procedure. Let U be the base preceding the mutation, and X and Y be the mutant and wild type alleles at a segregating site. Within the 100 bp around the mutation, we searched for the nearest sequences UX and UY, and we measured, in that context, the height of peaks X (*hc*_X_) and Y (*hc*_Y_). These values were then compared to the actual peak heights at the polymorphic site, X (*hp*_X_) and Y (*hp*_Y_). The frequency of the mutant Y was calculated as,

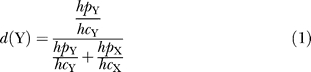



**Figure 3:**
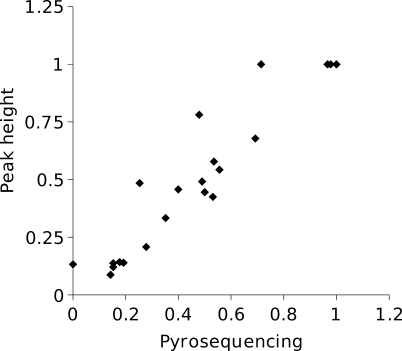
Estimated Mutation Frequencies for Cases in Which the Frequency Was Measured Both by Pyrosequencing and from Sequence Trace Peak Height

Where possible, the correction was carried out on both strands, and the frequencies averaged. In one case in which a variant was a 2-bp insertion in a microsatellite rather than a point mutation or a single-bp indel, the height of the first polymorphic base was used to estimate the mutation frequency. For mutations detected by direct sequencing, the frequency of the mutation was estimated as above, and not by pyrosequencing. We checked the performance of the above method on mutations whose frequencies were estimated both by pyrosequencing and from peak height on a sequence trace. The correspondence between the estimates (Figure 3) is generally very good (slope = 0.93; *r* ^2^ = 0.93).

### Inference of the mtDNA mutation rate and effective population size.

We assumed that mutations appear in the mitochondrial genome at a rate μ per site per generation, that μ is sufficiently low that multiple mutation events at the same site can be ignored, and that the fates of new mutations are determined solely by genetic drift. Under a neutral model, the fixation rate at equilibrium between drift and mutation is proportional to the mutation rate [13]. The probability of ultimate fixation in a maternal lineage of a mutation *i* that occurred at some time in the past is proportional to its current frequency within an individual (*d_i_*). An estimate of the mtDNA mutation rate per fly generation after *t* generations of MA can therefore be obtained from

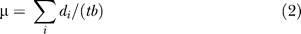
where *b* is the total number of mtDNA bases scanned, summed over MA lines, and the summation is over the mutation events detected. Within a set of MA lines of a given genotype, *t* was essentially invariant, and was assumed to be a constant. This assumes that all mutations have been detected, regardless of their frequency.

We also jointly estimated μ and the effective number of mitochondrial genomes transmitted per female per generation (*N*_e_) by ML. In our ML approach, we actually estimate the fraction of unmutated sites (*f*_0_), and from this we estimate μ. We allow for error variation in the observed allele frequencies by modelling this as a truncated normal distribution, bounded by 0 and 1. The time to coalescence of mitochondrial genomes sampled from different individuals within a line was assumed to be negligible compared to the time scale of the MA experiment and the time to coalescence of mitochondrial genomes within an individual. Males are thought to contribute a negligible proportion of mitochondrial genomes to the zygote [43,44]. In females, the drift process will depend on the number of mitochondrial genomes transmitted each germ-line mitosis and during meiosis, and this will be subject to variation. We modelled this drift process by a single parameter, *N*_e_, which is inversely proportional to the drift experienced by the population of mitochondria within an individual each fly generation [17].

By transition-matrix methods, we generated the expected allele-frequency distribution for mutations that appeared during *t* generations of MA. We equate *N*_e_ to *N*, the size of the population modelled by the transition matrix. For a mutation appearing at a frequency of 1/*N* at generation 0, we calculated the vector, **u**(*t*), that specifies the probabilities of the population having an allele frequency of *j*/*N* (0 ≤ *j* ≤ *N*) at generation *t* using a haploid Wright-Fisher transition matrix, assuming no selection [45]. The cumulative, unscaled allele-frequency probability vector (**v**′) for mutations that occurred in generations 0,..,*t* is then

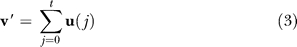



The vector **v**′ contains the unscaled cumulative frequency distribution of mutations that have actually occurred, but does not include a contribution from sites that remained unmutated over the whole experiment. The frequency of these sites, *f*_0_, is a parameter that we estimate in the model, and was incorporated into the scaled cumulative frequency distribution vector, **v**, which is used in the likelihood computations. Writing Σ**_v_**_′_ = 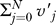 the elements of **v** are then scaled as follows:

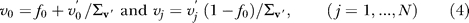



The data consist of a vector **d** of estimated allele frequencies at *m* segregating or mutant fixed sites and the number *n* of wild-type fixed sites. We assumed that the allele frequencies at the wild-type fixed sites were measured without error. At the segregating and fixed mutant sites, we assumed that the observed frequencies were subject to measurement error. To model this, we assumed that frequencies have a truncated normal distribution about their expectation, where φ(*x*|*M*, *V*_E_) is the probability density function for the untruncated normal distribution of mean *M* and variance *V*_E_ *.* The overall likelihood is the product of likelihoods of the observed frequencies (*d_i_*) of the *m* segregating or fixed mutations. This is the weighted sum, over all possible allele frequencies in the population of size *N*, of the probability density of the scaled normal distribution at point *d_i_*, given that the mean allele frequency is *j*/*N* and the error variance is *V*_E_:

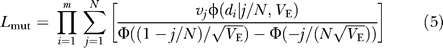
where Φ(*y*) is the cumulative probability function for the standard normal distribution from –∞ to *y*, evaluated as described by Abramowitz and Stegun [46]. The denominator of Equation 5 truncates the normal distribution at 0 and 1, since frequencies outside this range cannot occur, and allocates the missing density proportionately. The likelihood for the wild-type fixed sites is simply the product, over the *n* unmutated sites, of the probability of a site being fixed for the wild type allele, i.e.,

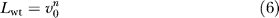
and the overall log likelihood was log(*L*_mut_) + log(*L*_wt_).

The number of mutant sites was generally small, so in practice the frequency distribution often contained little information to estimate *V*_E_ reliably. This led to numerical problems, especially for the Florida line data for which the number of mutations detected was particularly small. To improve the stability of the inference procedure, we therefore penalised implausible *V*_E_ values using a likelihood function based on the error variance inferred from five mutations for which we had two independent replicate measures of allele frequency. Only a single randomly chosen observed frequency from each of these lines is used in evaluating Equation 5, in order that each mutation received equal weight in the estimation of μ and *N*_e_. The likelihood penalty function was the result of the following:

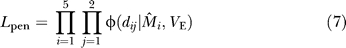
where *d_ij_* is the measured allele frequency for mutation *i*, replicate *j*, and **M̂*_i_* is the mean allele frequency for mutation *i*. The overall log likelihood was then log(*L*_mut_) + log(*L*_wt_) + log(*L*_pen_), and was maximized as a function of the three parameters of the model, one discrete (*N*) and two continuous (*f*_0_ and *V*_E_). For all possible fixed values of *N* between wide limits, we maximized log likelihood as a function of *f*_0_ and *V*_E_ using a combination of a grid search and the simplex algorithm [47,48]. The global ML and its associated maximum likelihood estimates correspond to the value of *N* giving the highest log likelihood. Because **v** is scaled such that it contains contributions from *N* mutations each generation accumulated over *t* generations, the MLE of the mutation rate per site is

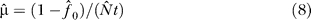
where *f̂*_0_ and **N̂** are MLEs. The estimate of the effective population size of mitochondrial genomes is simply *N*_e_ = *N̂*.

### Simulations.

We investigated the performance of the ML procedure by simulation. For simulation parameters *N*, *t*, and μ, we generated *t* + 1 cumulative allele-frequency probability vectors (**v**) corresponding to generations 0,..,*t* of MA, as described above. In each simulation run, a number of mutations was sampled from a Poisson distribution with parameter μ*tNs*, where *s* is the total number of sites simulated (100,000 in the cases considered). For each mutation, we randomly sampled one of the *t* + 1 allele frequency vectors with replacement, and from it randomly sampled an allele frequency with probability proportional to its density in the frequency vector. To this frequency we added a normal deviate with mean 0, variance *V*_E_. In order to model a truncated normal distribution of frequencies, deviates were rejected until the resulting frequencies lay in the range 0…1. We analysed each simulated dataset assuming the value of *t* simulated, while estimating *N*, μ, and *V*_E_ as unknowns. Additionally, to assess the amount of bias induced by failing to experimentally detect low-frequency mutations, we ran simulations in which sites with mutant frequencies <*k* were excluded from the simulation output.

## Supporting Information

Table S1 Regions of the D. melanogaster Mitochondrial Genome Scanned by DHPLC and Direct Sanger Sequencing (89 KB PDF)

Table S2 Details of Mutations in Florida Lines Detected by DHPLC, but Not by Direct Sequencing, and Vice Versa (85 KB PDF)

Table S3 Primers Used for PCR Amplification in Mutation Scanning by Direct Sequencing and DHPLC (56 KB PDF)

Protocol S1 DNA Sequencing Traces Showing Mutants and Wild Types (537 KB PDF)

## Abbreviations

DHPLC: denaturing high performance liquid chromatography
indel: insertion-deletion
mtDNA: mitochondrial DNA
MA: mutation accumulation
ML: maximum likelihood
