# Supplementary material for: Direct Estimation of the Mitochondrial DNA Mutation Rate in Drosophila melanogaster
Source: PLoS Biol. 2008 Aug 19;6(8):e204. doi: 10.1371/journal.pbio.0060204 (PMC2517619; doi:10.1371/journal.pbio.0060204)

Haag-Liautard et al.

Direct estimation of the mitochondrial DNA mutation rate in *Drosophila melanogaster*

Supplementary Material

Position: 1,675

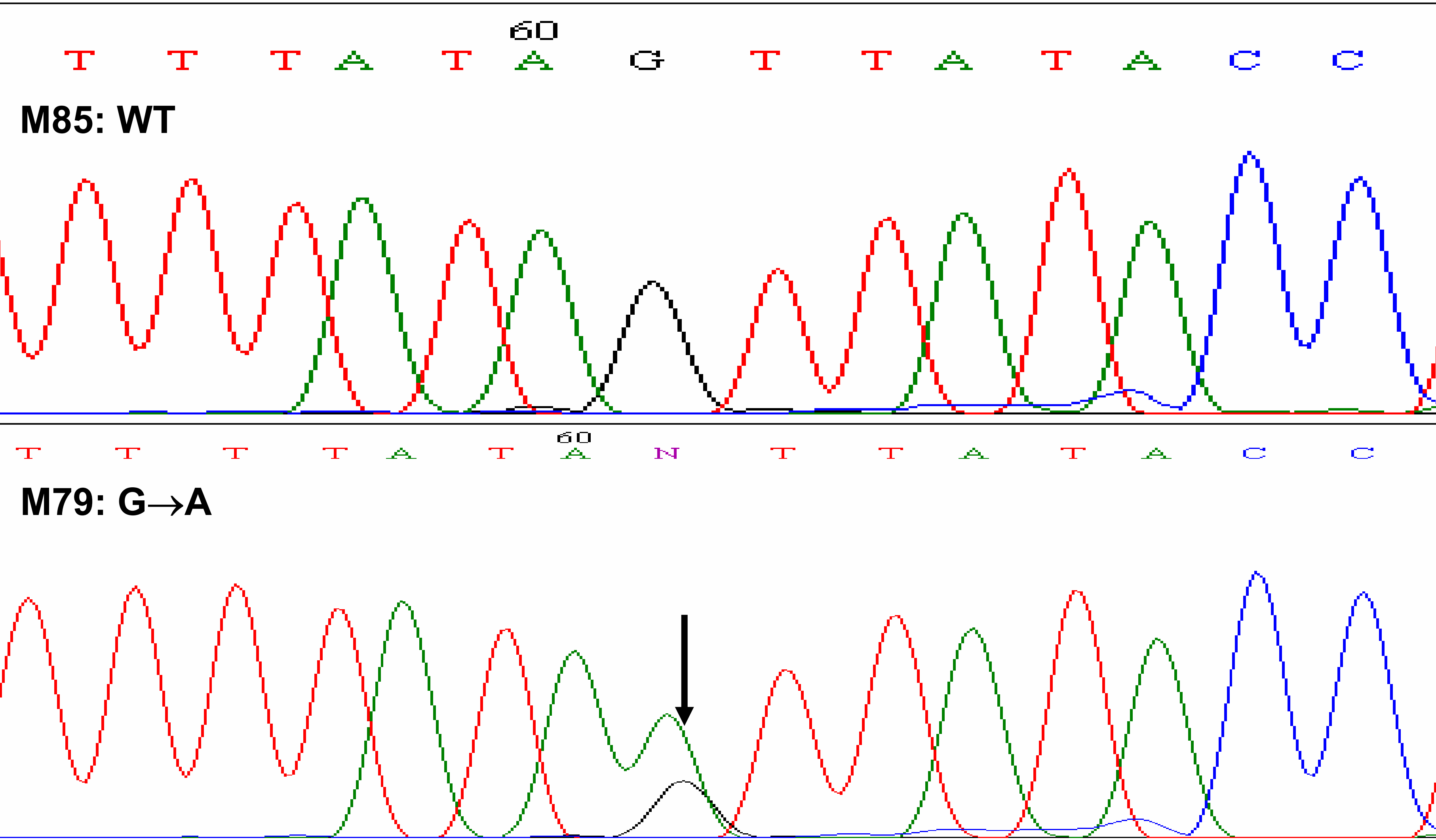

Position: 2,693

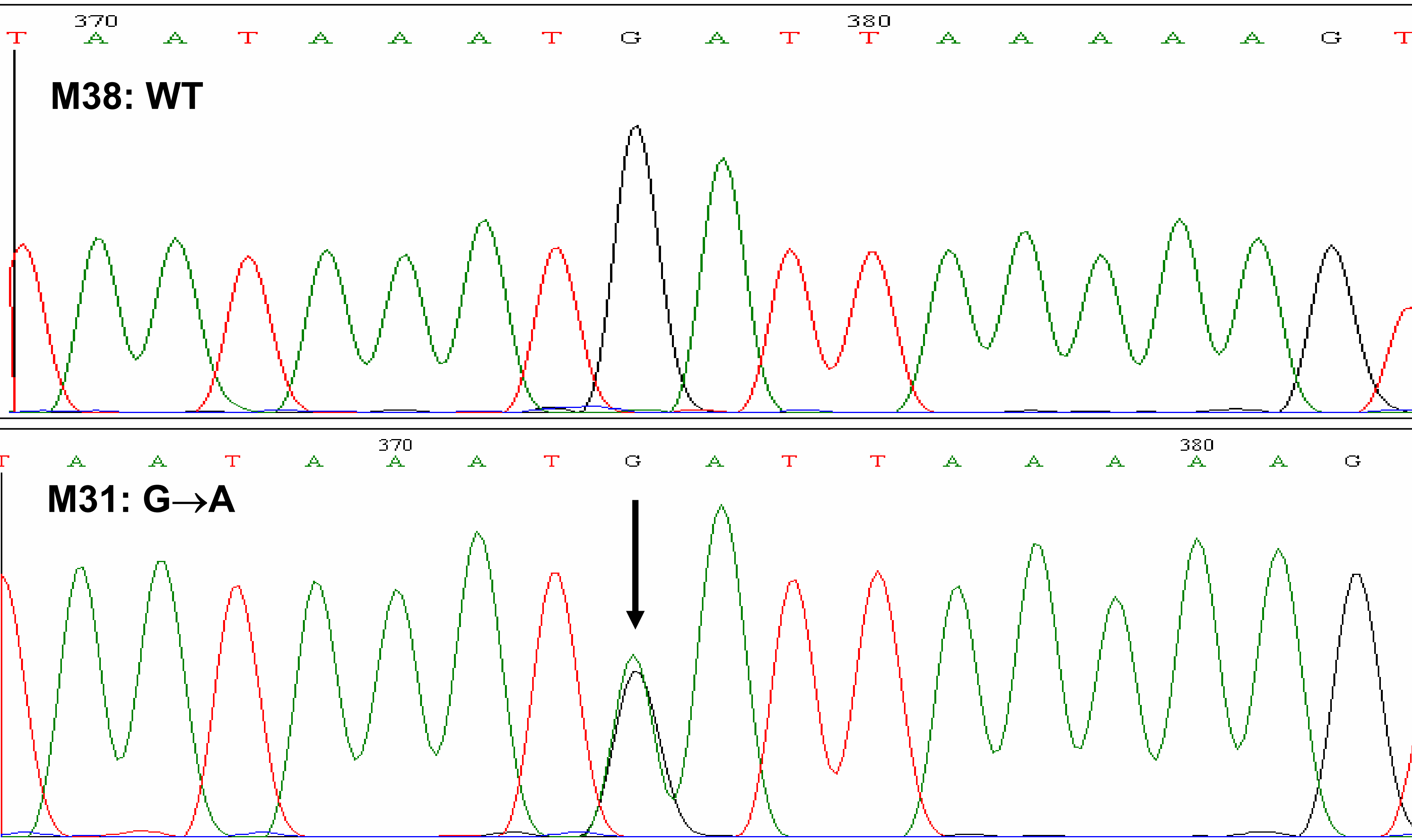

Position: 2,875

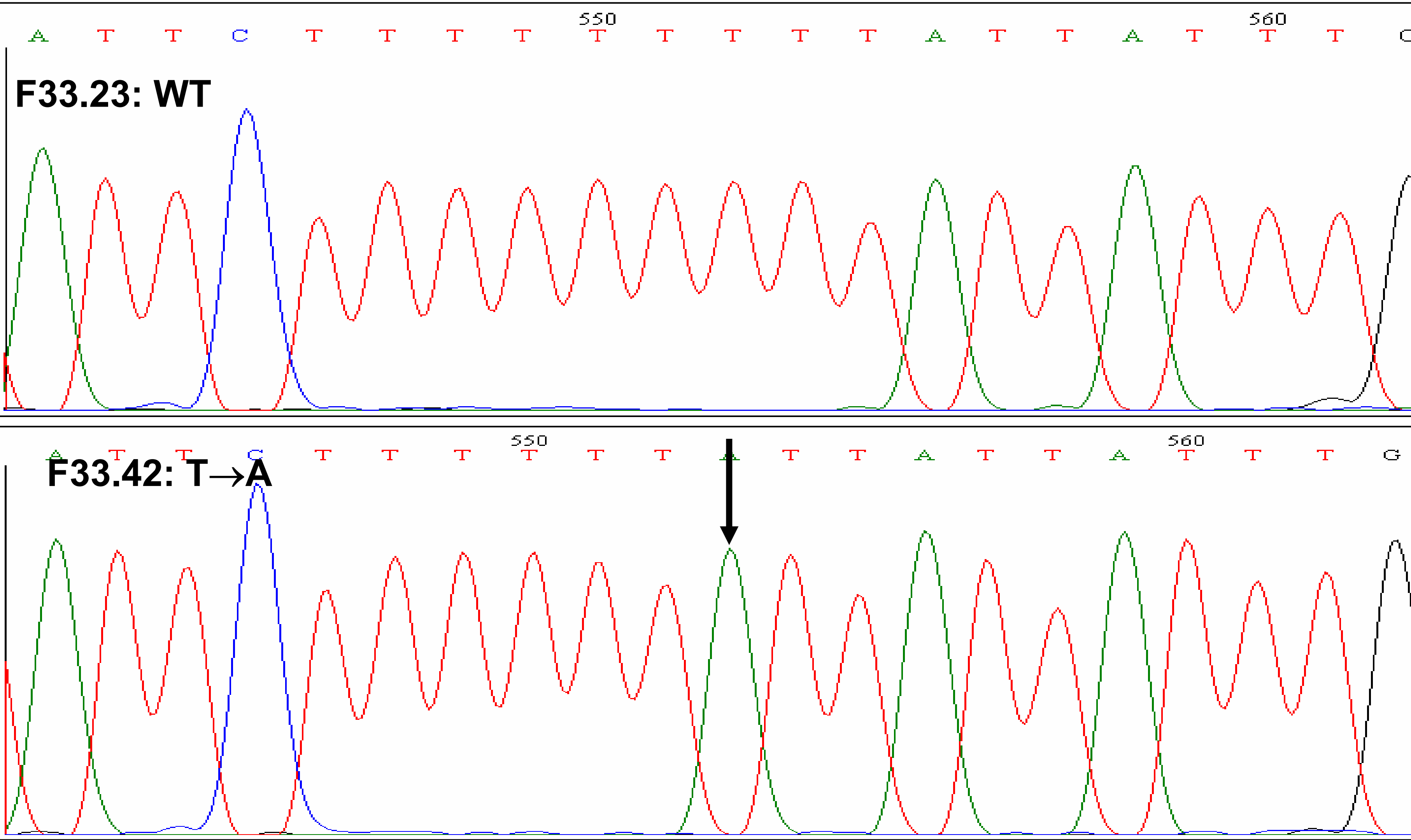

Position: 2,877

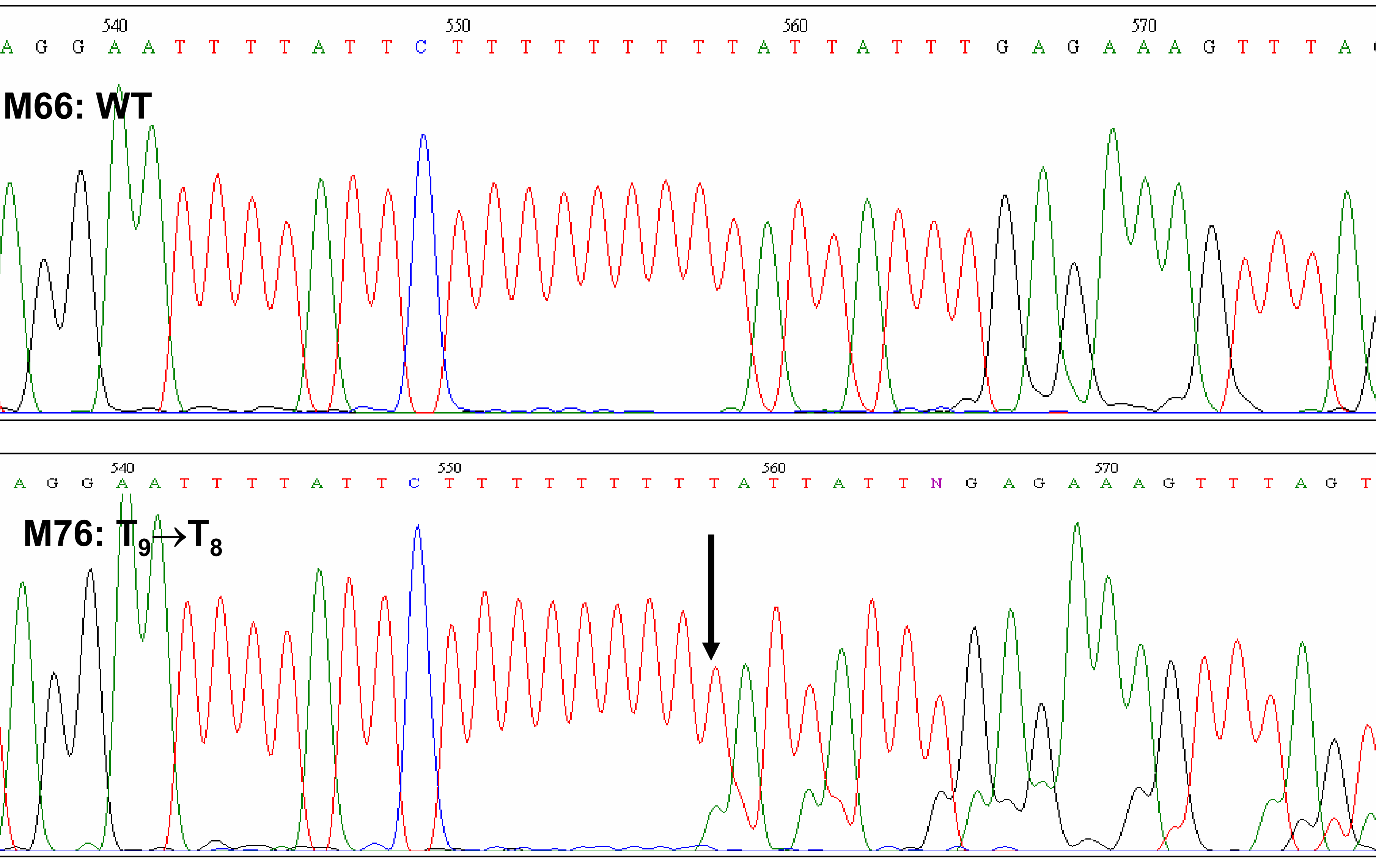

Position: 2,877

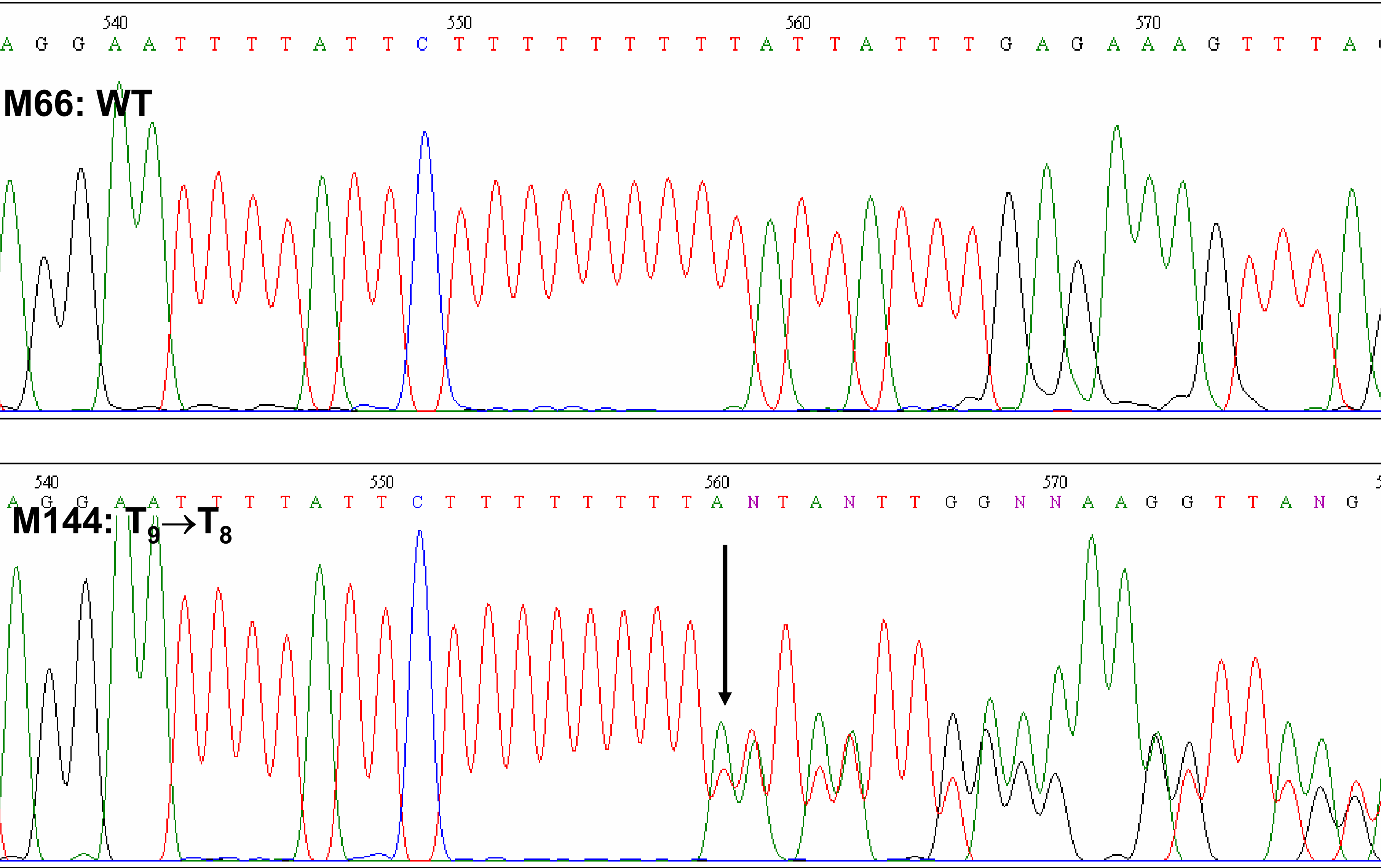

Position: 3,254

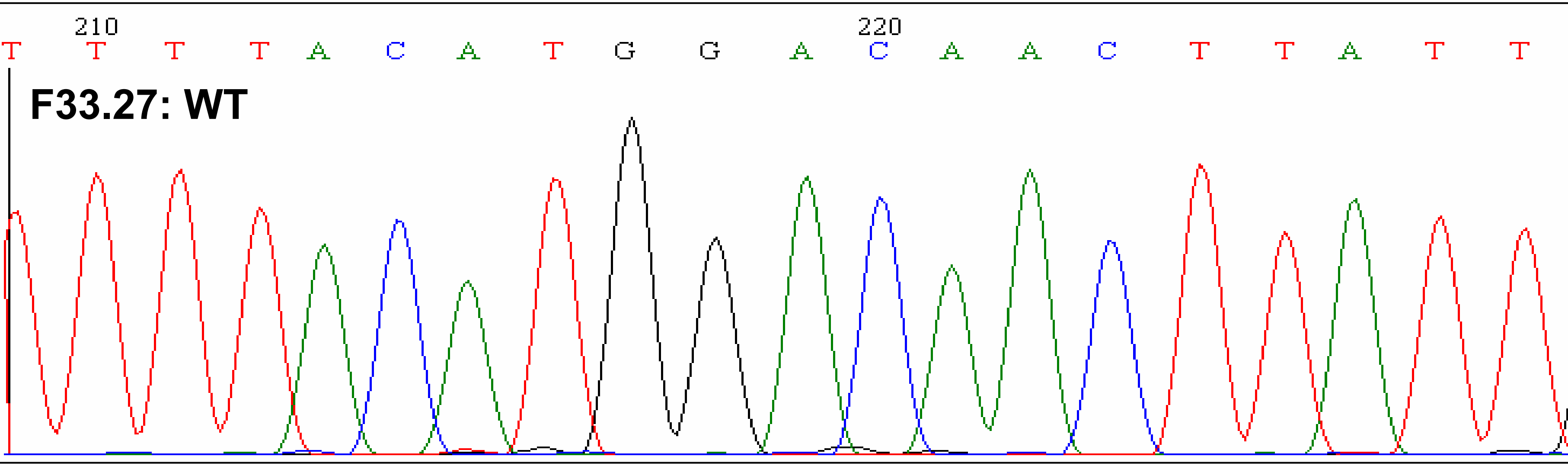

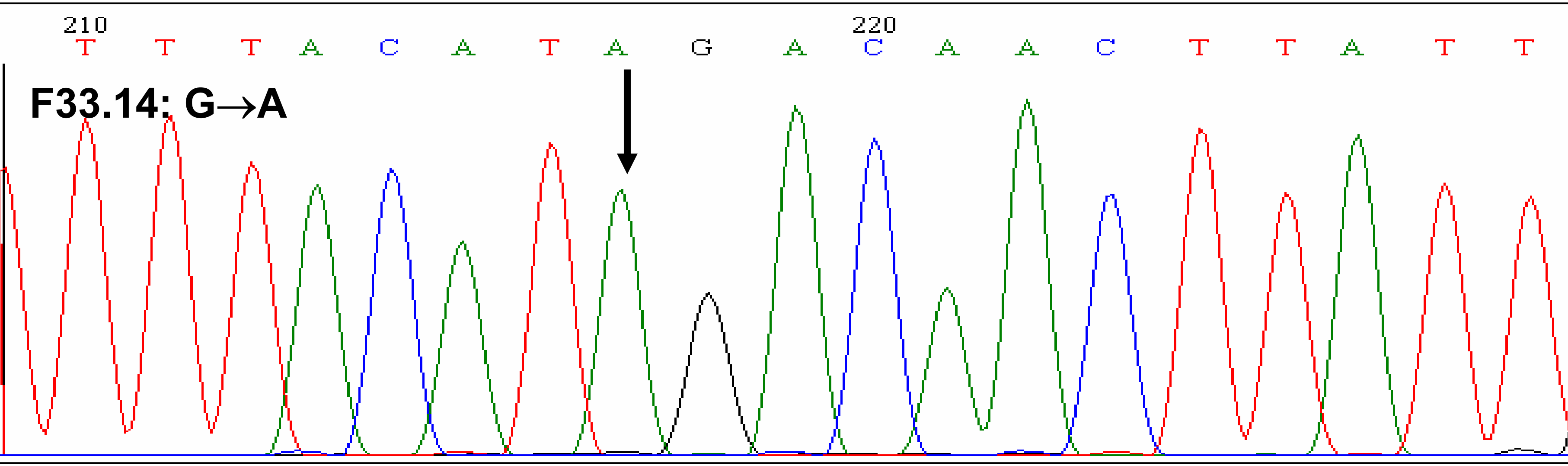

Position: 4,098

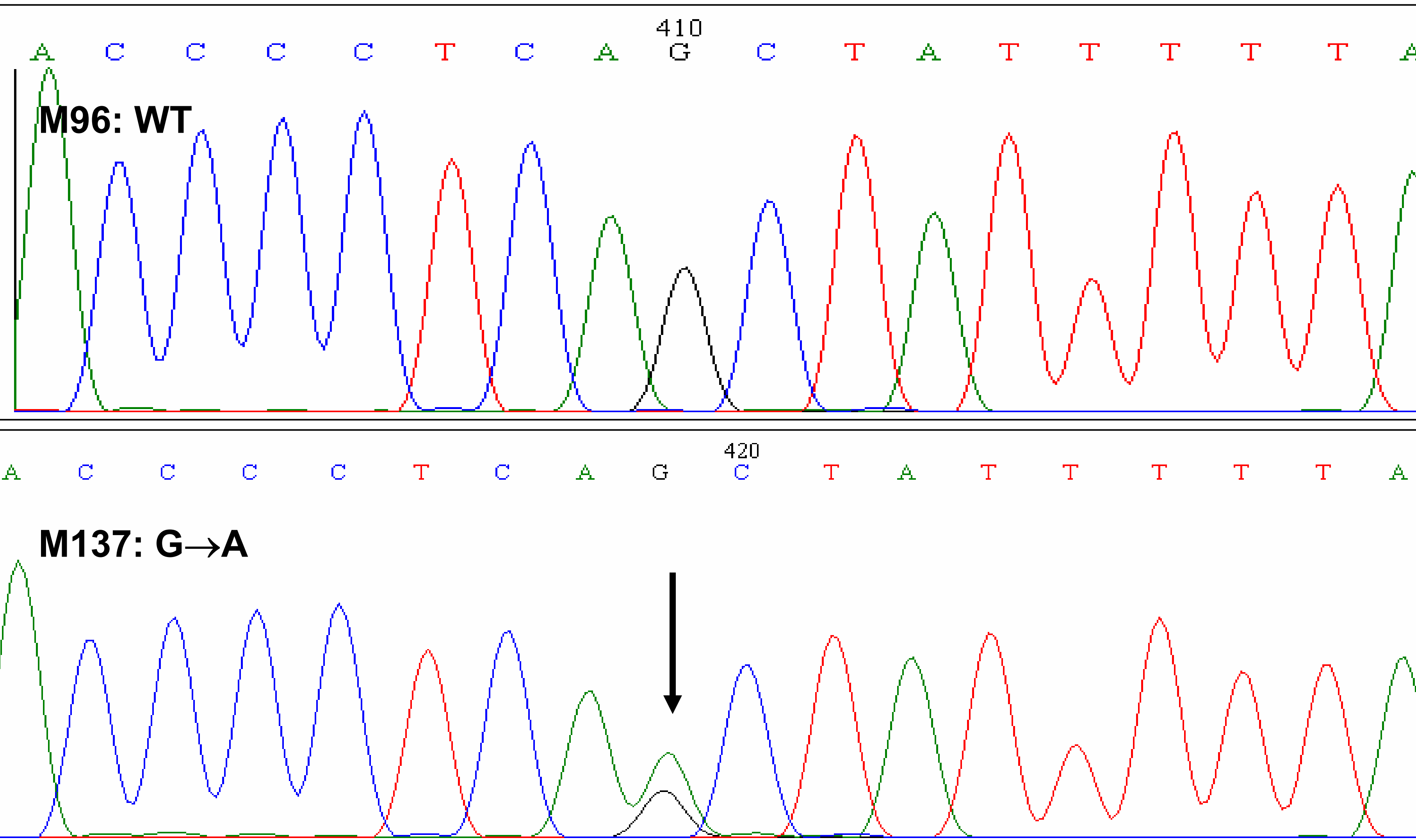

Position: 4,308

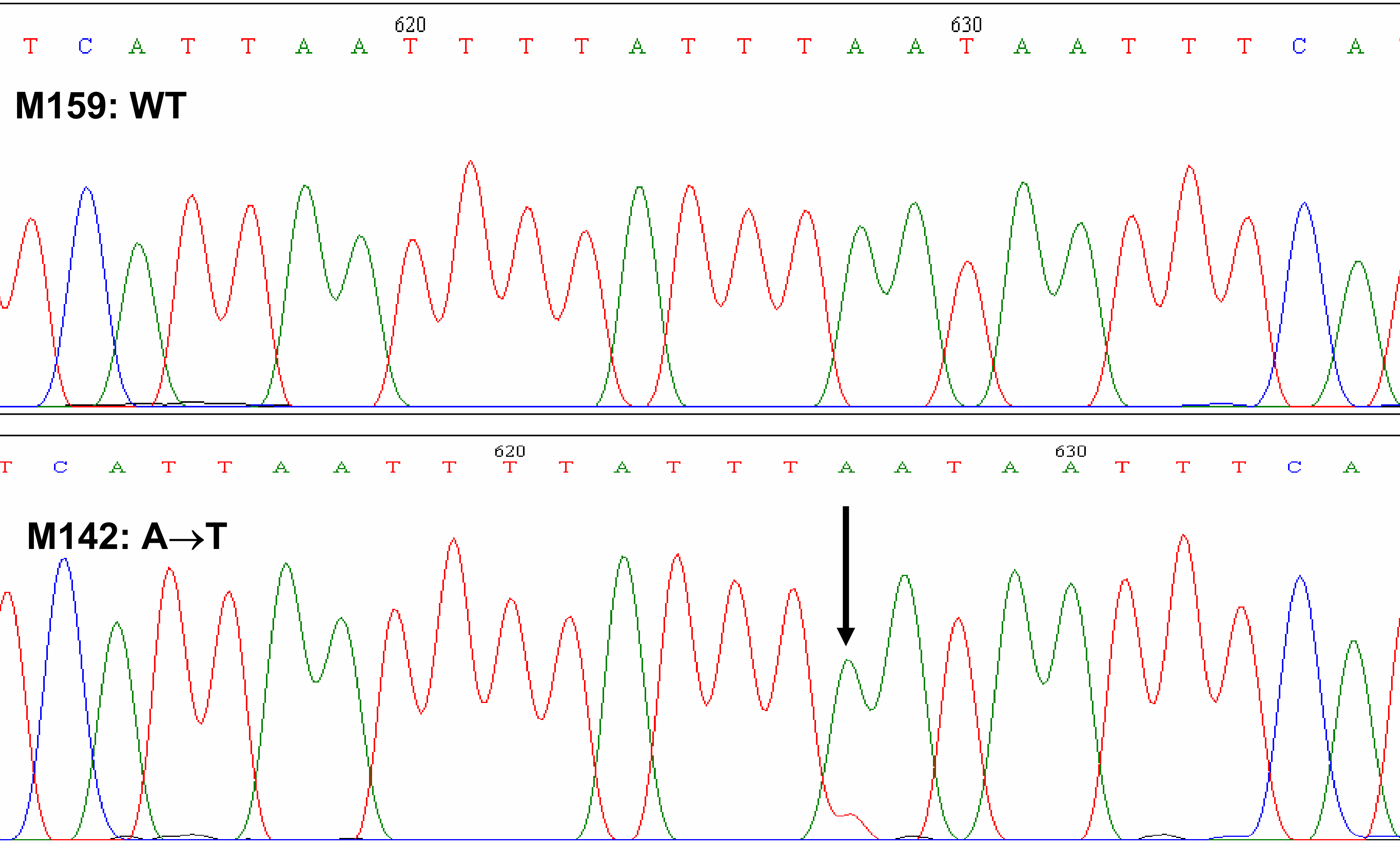

Found in two independent PCRs/sequences

Position: 4,321

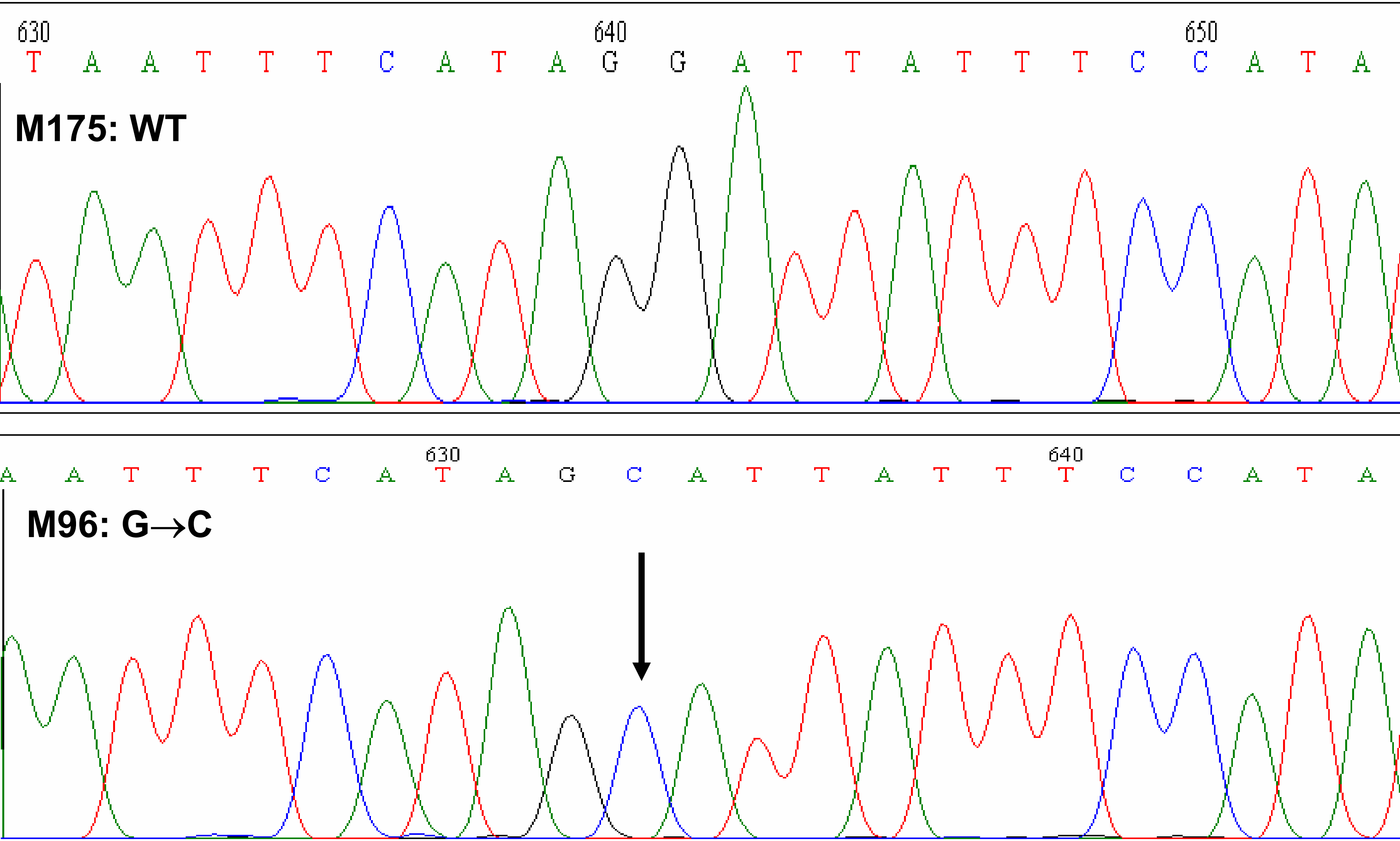

Position: 4,459

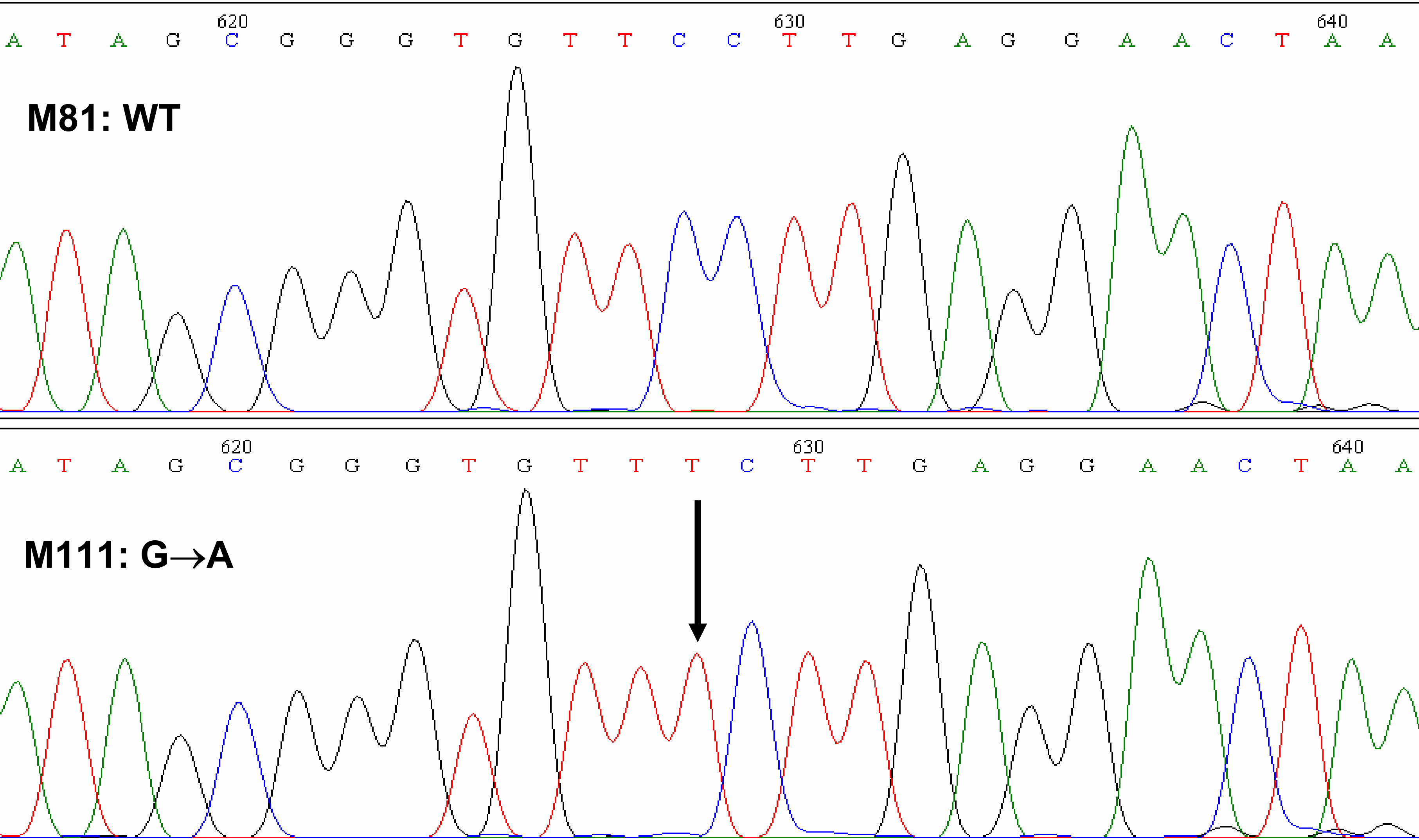

Mutation visible only on reverse sequence

Position: 4,533

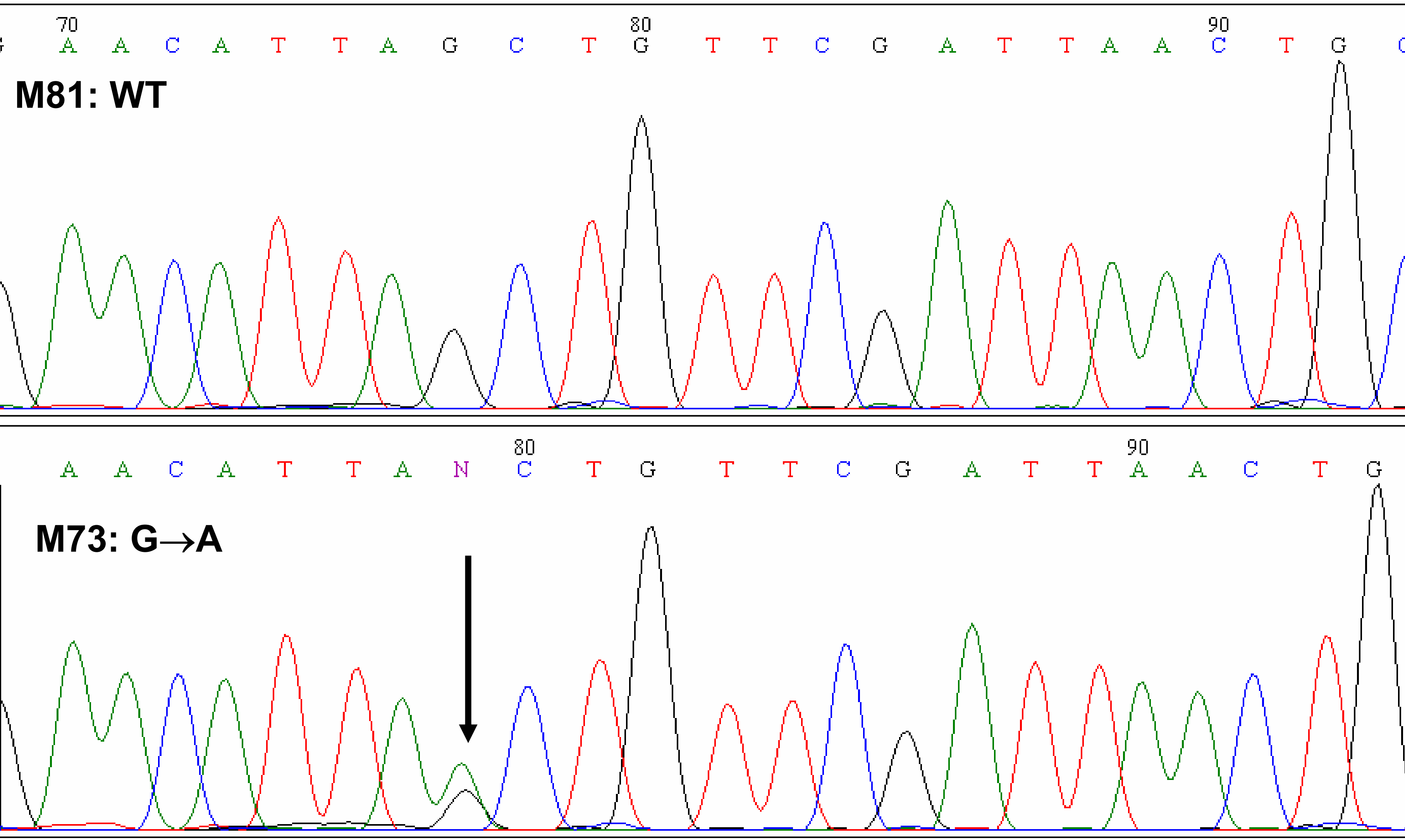

Position: 4,588

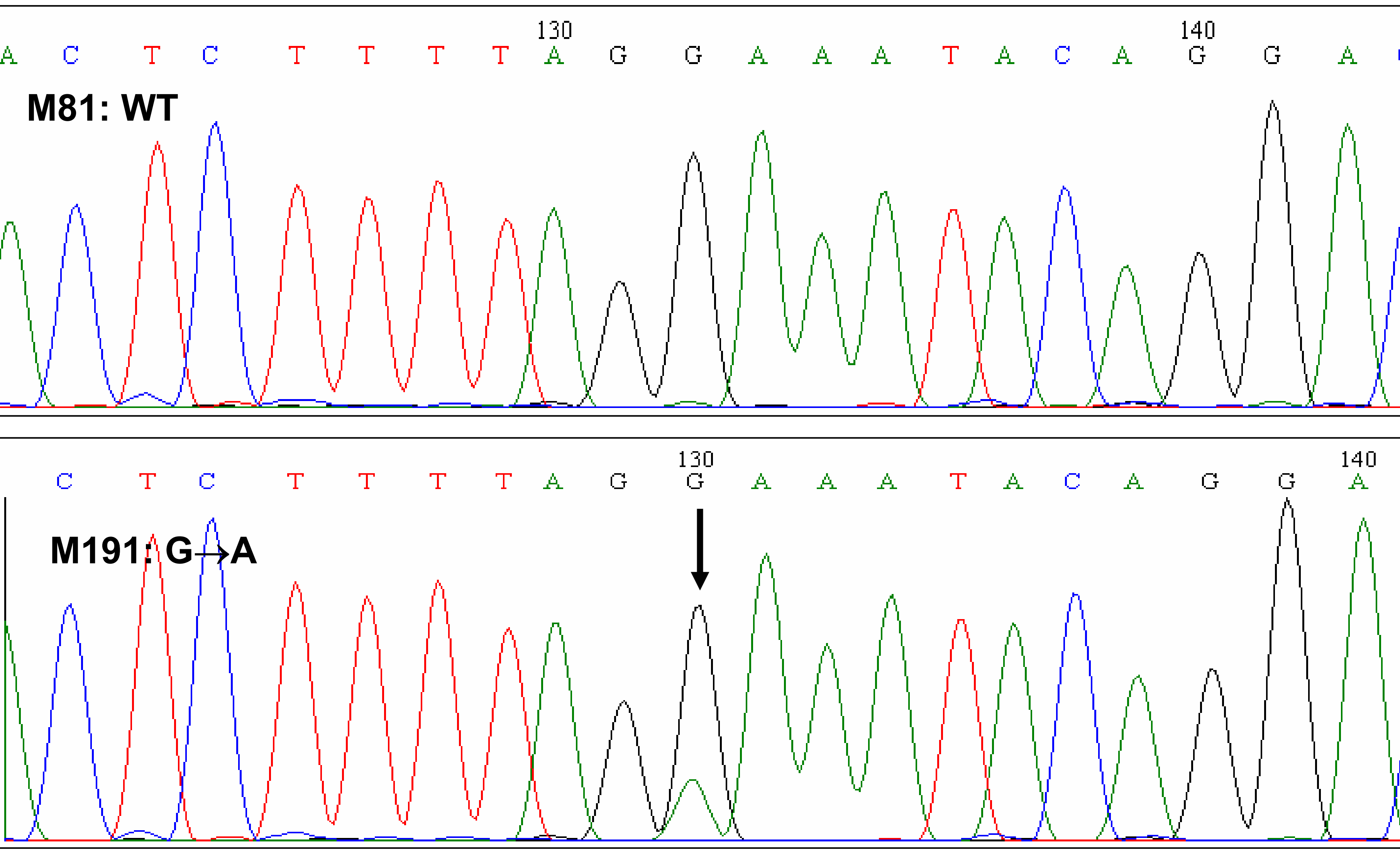

Position: 4,708

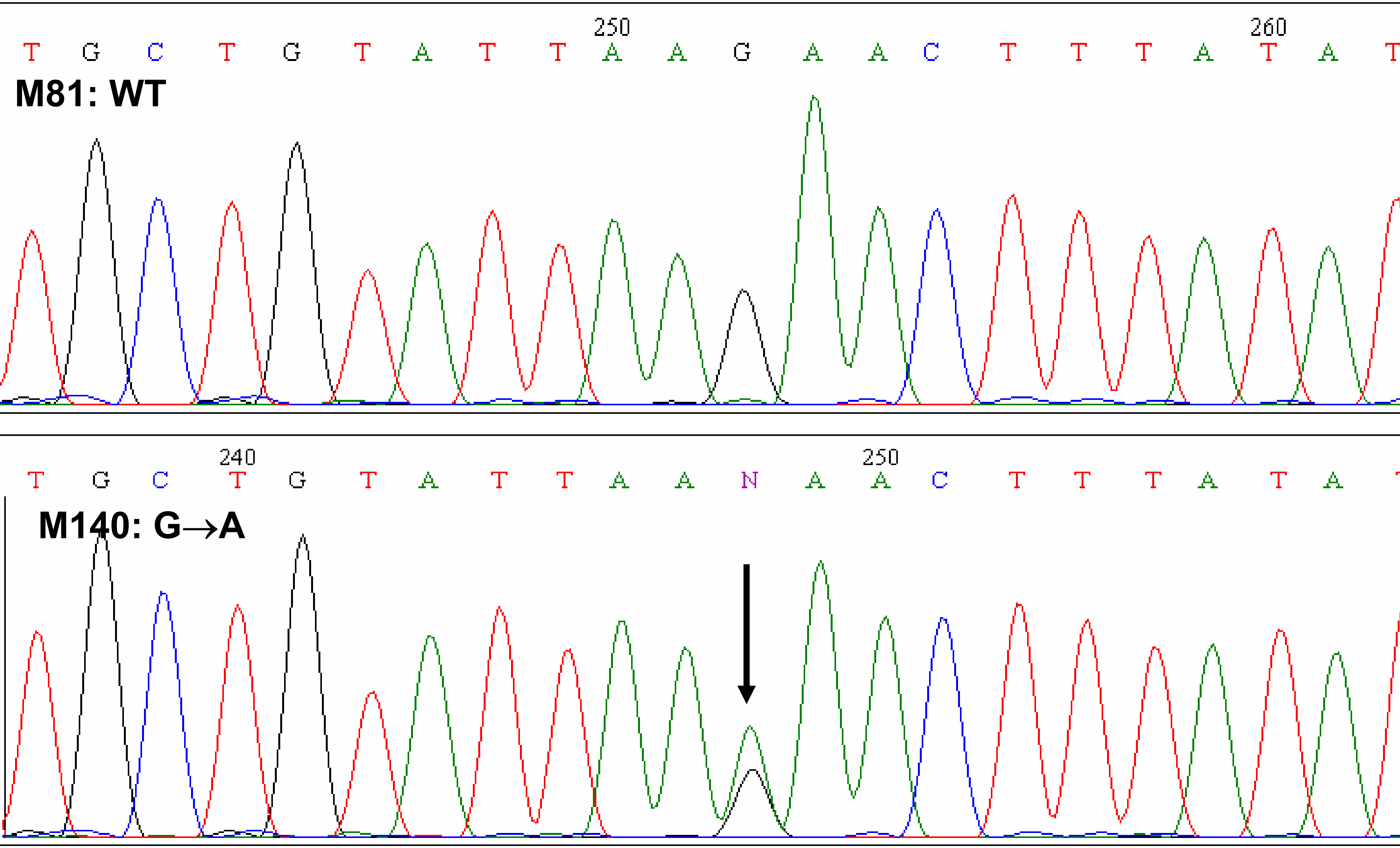

Position: 5,187

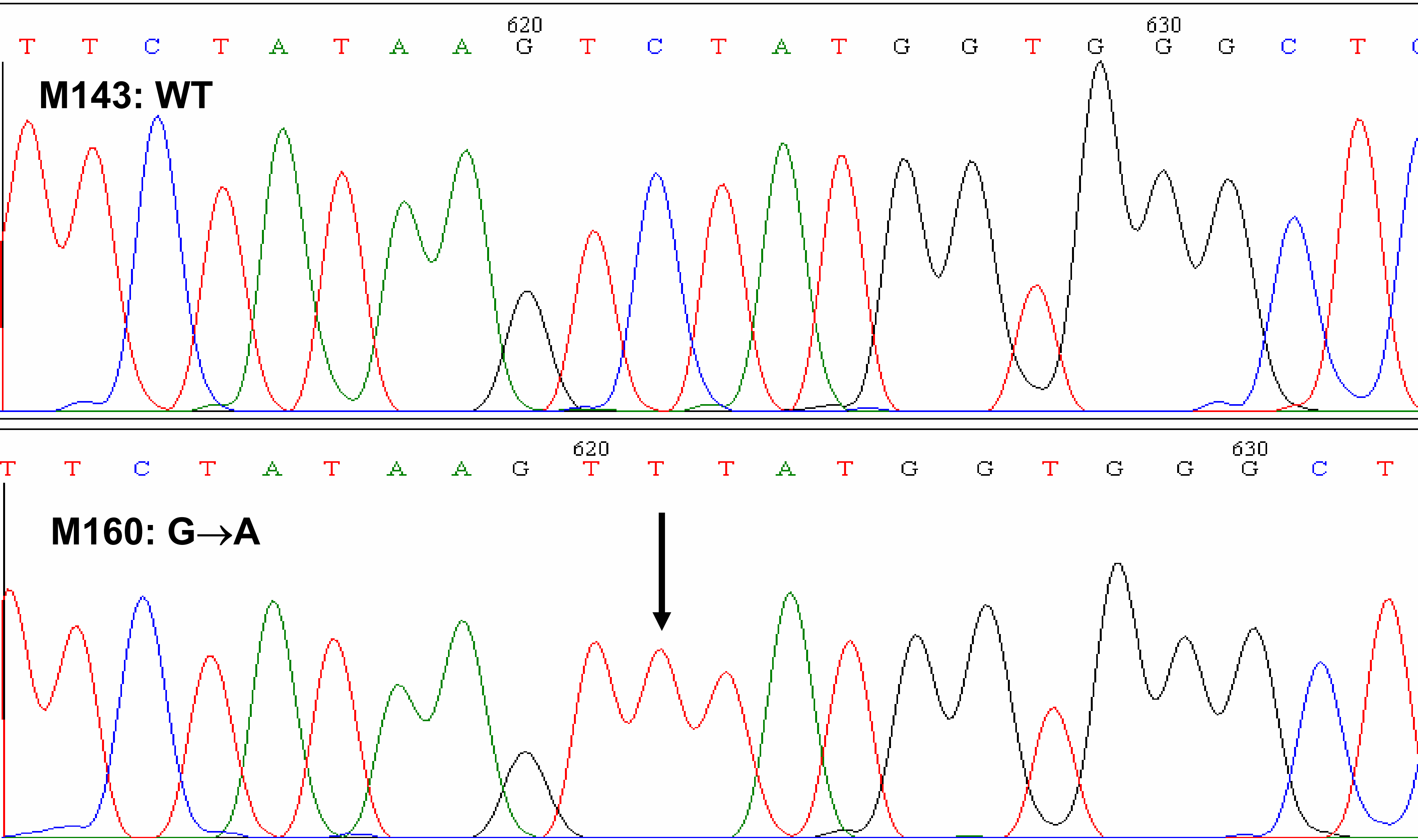

Mutation visible only on reverse sequence

Position: 5,970

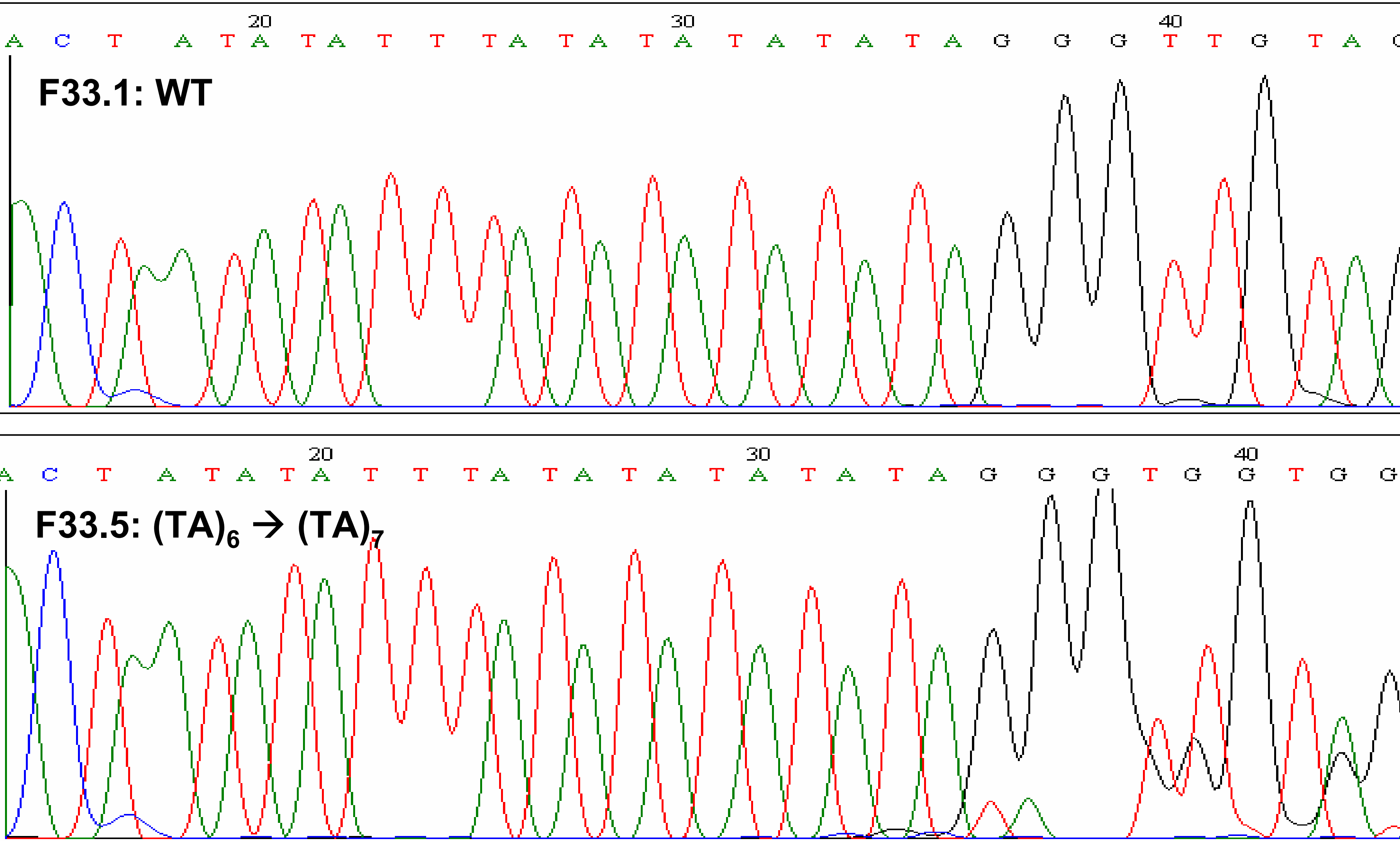

Position: 5,970

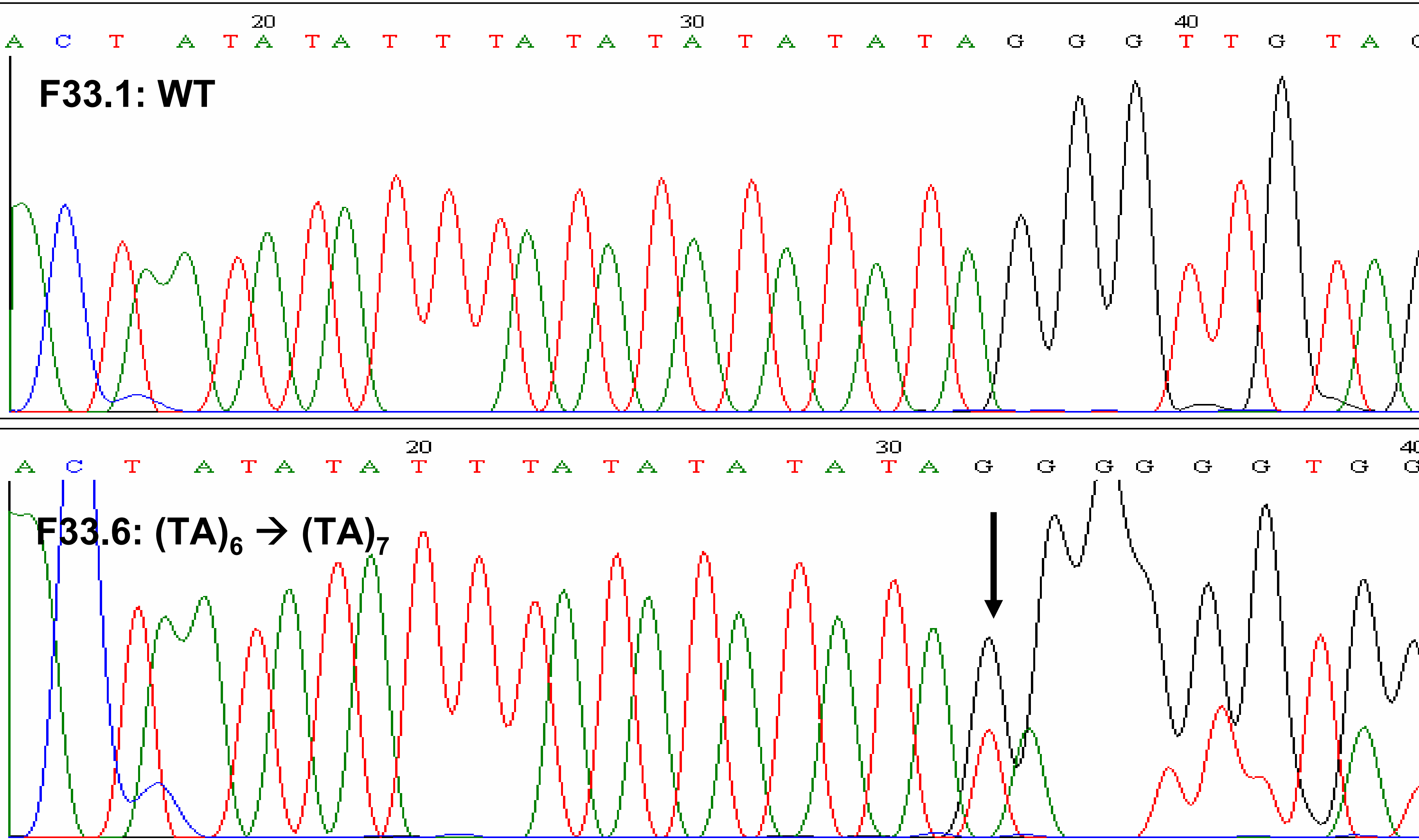

Position: 5,970

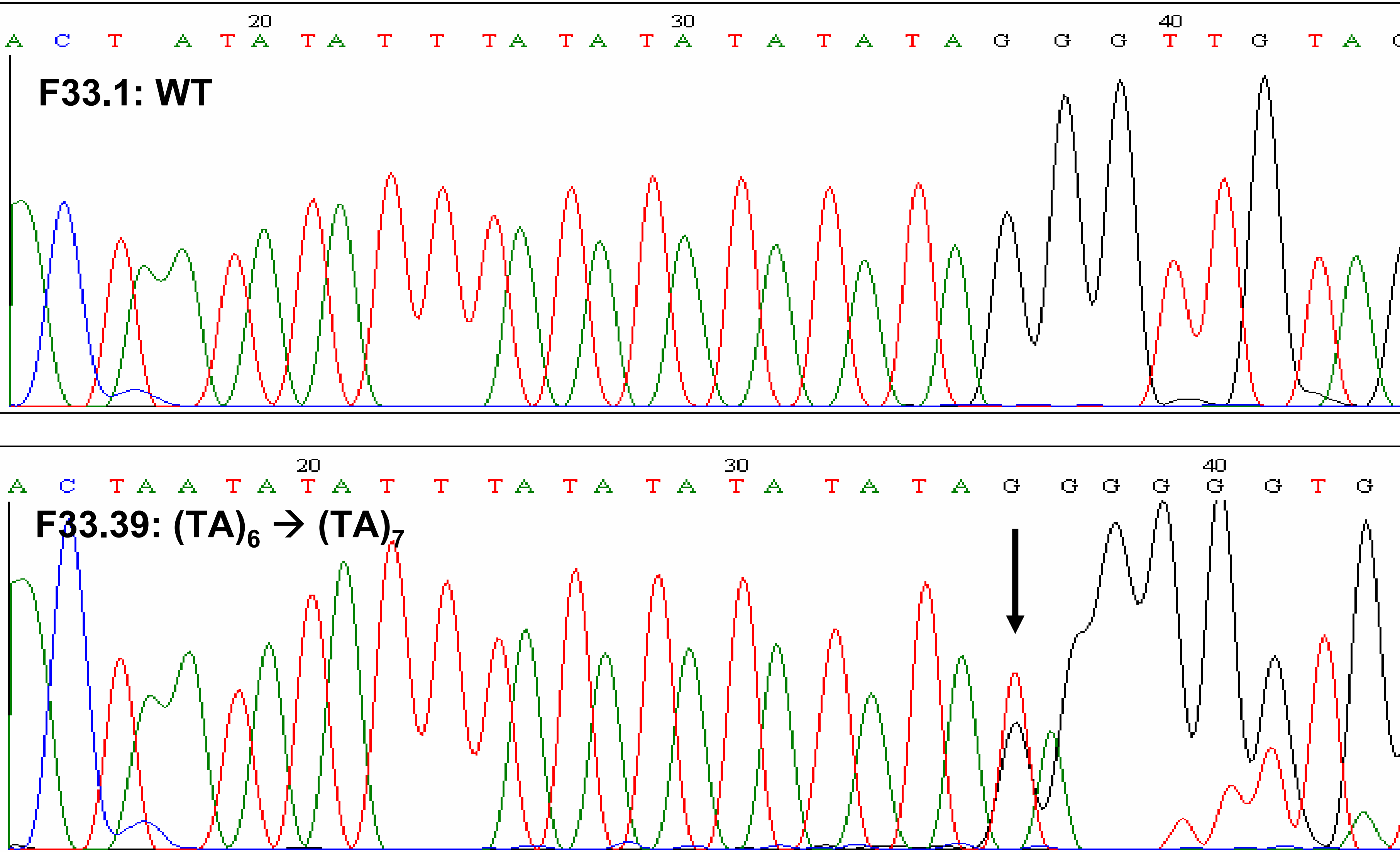

Position: 5,970

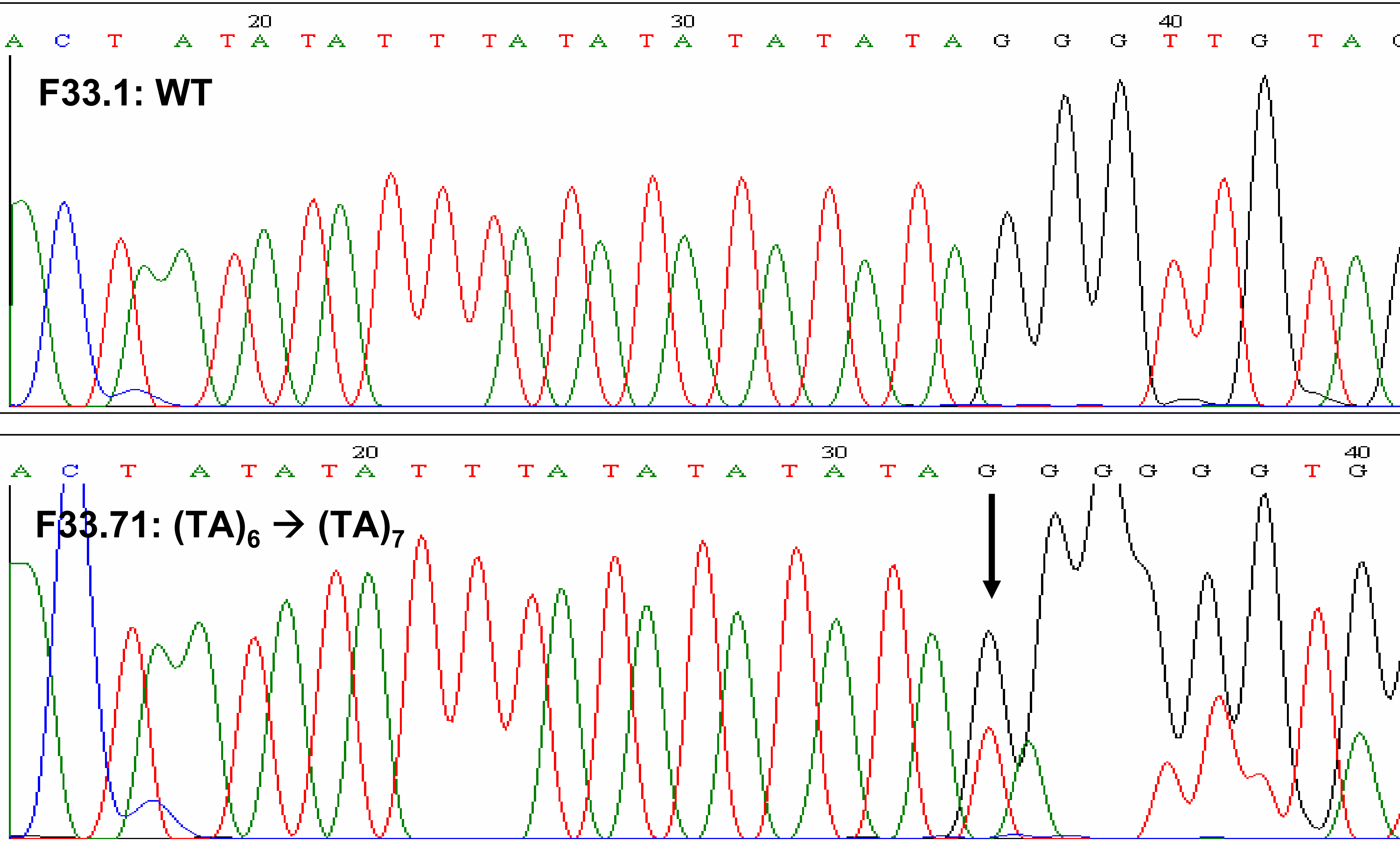

Position: 5,970

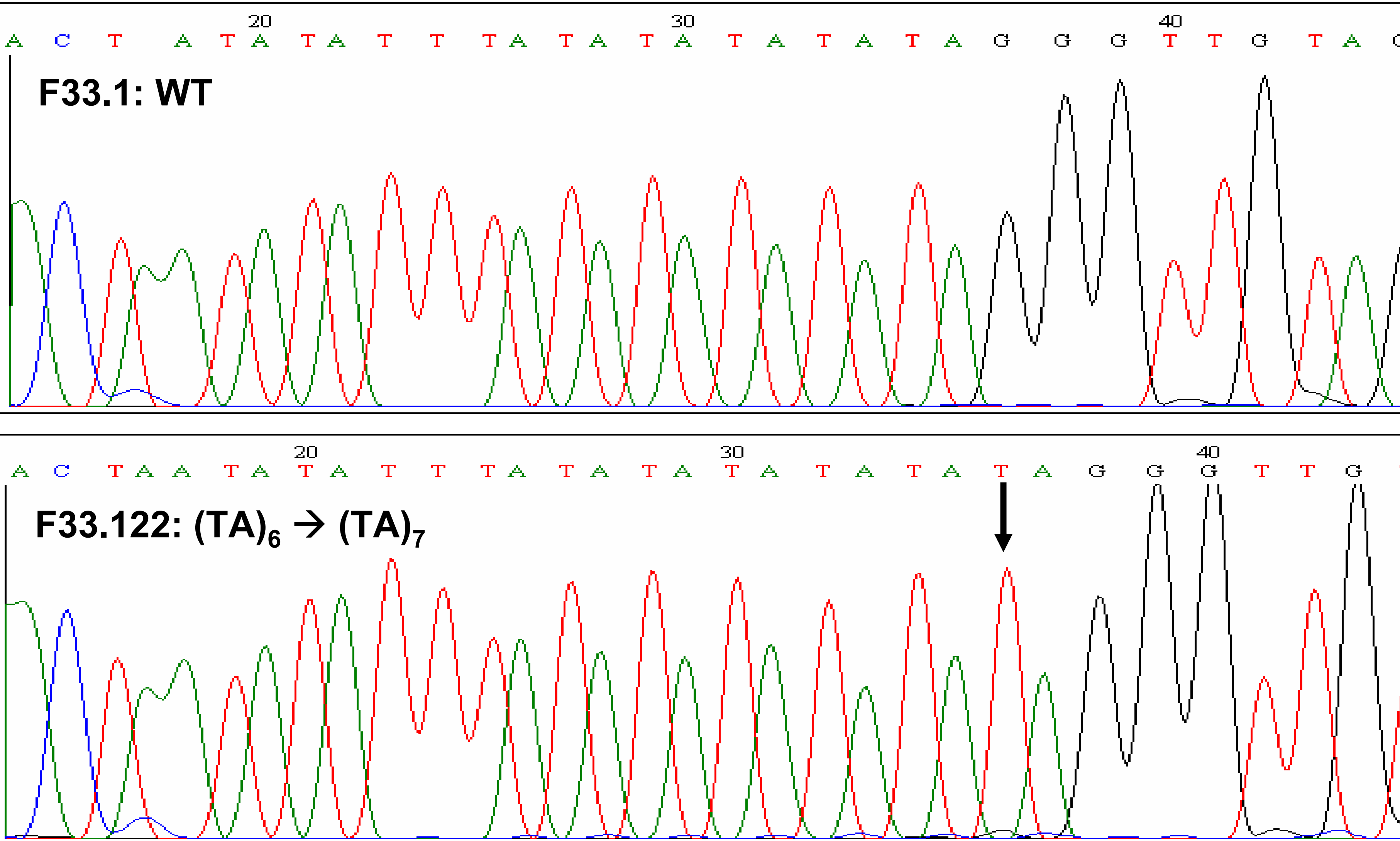

Position: 7,125

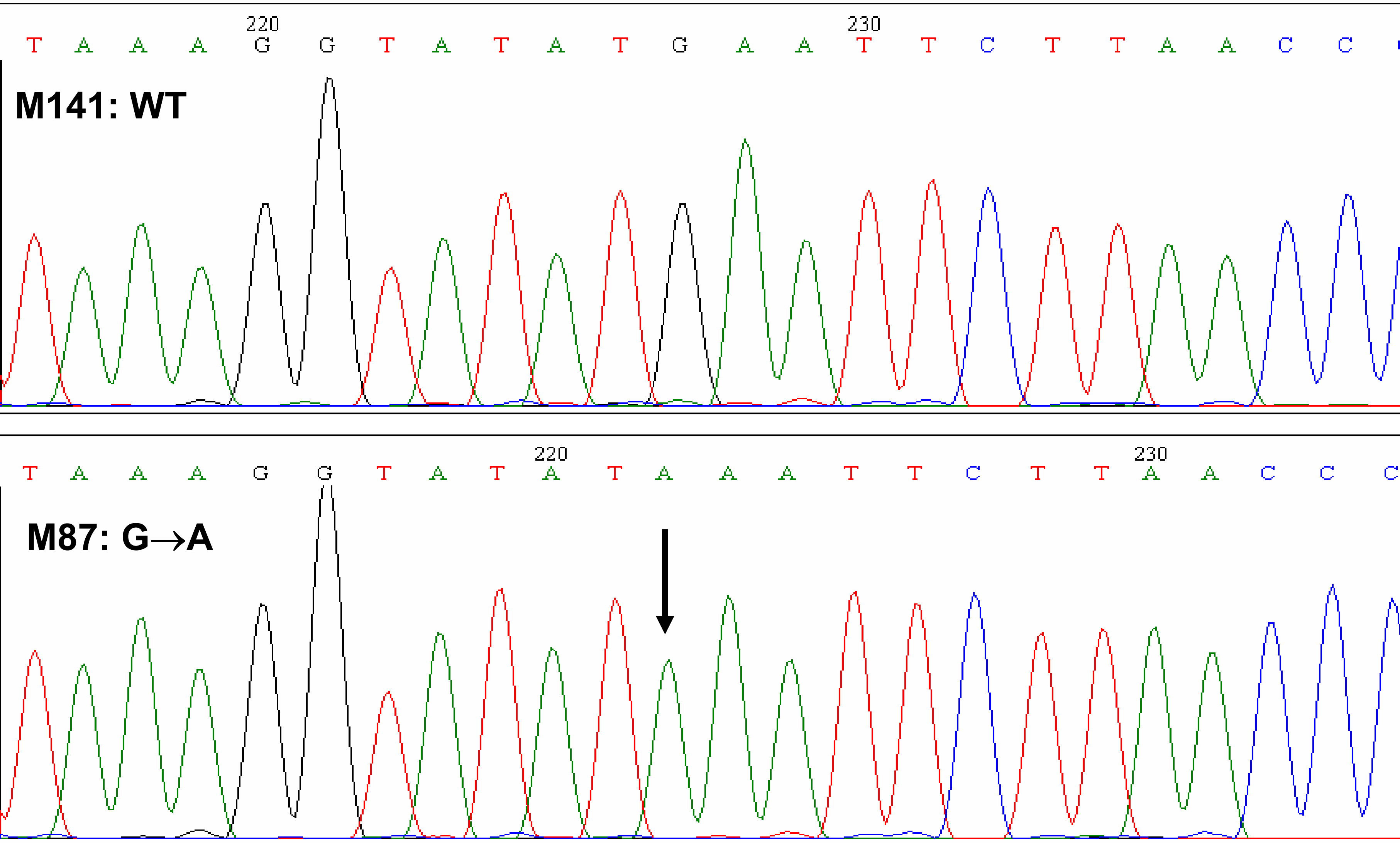

Position: 7,382

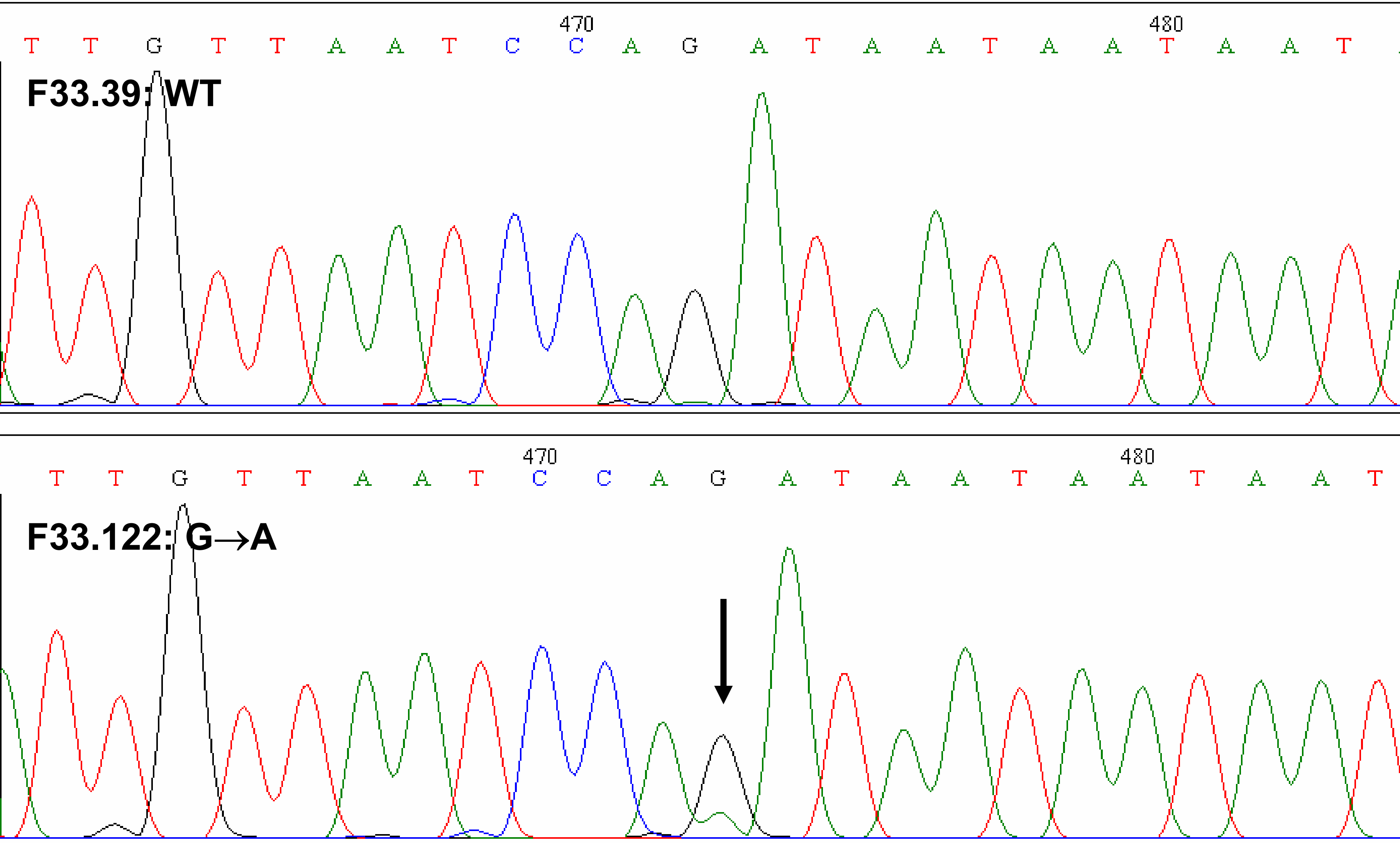

Position: 7,415

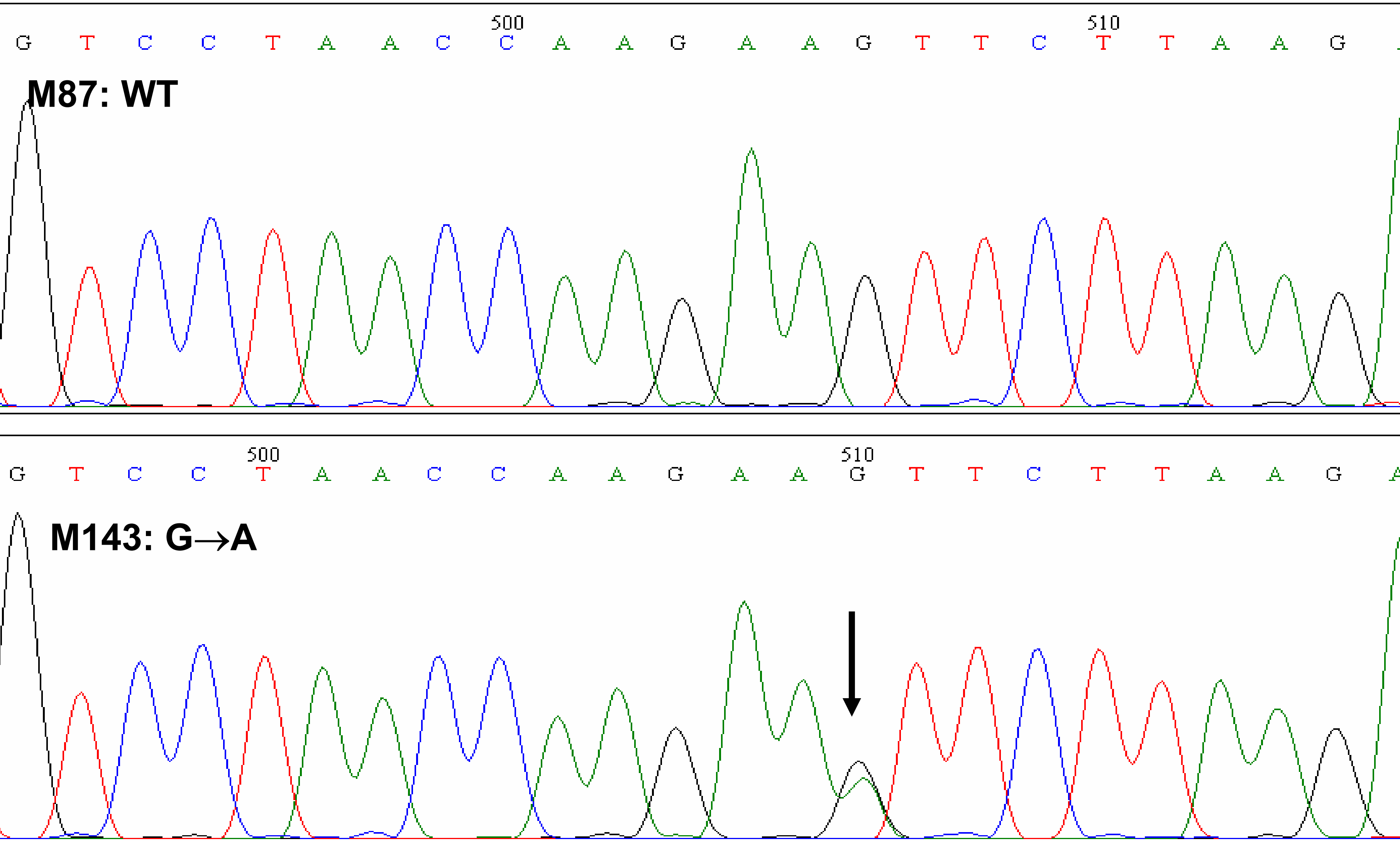

Position: 8,093

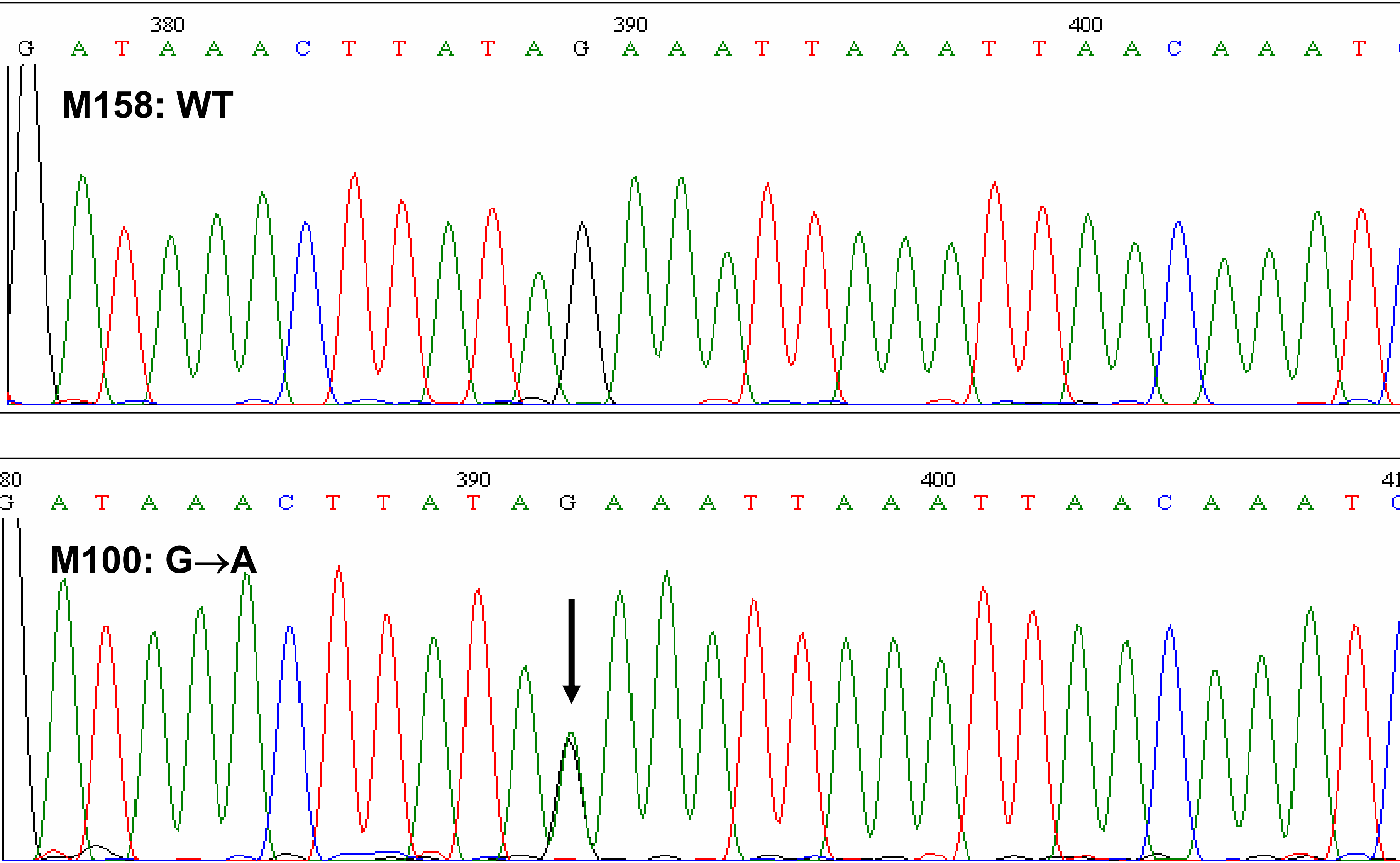

Position: 8,192

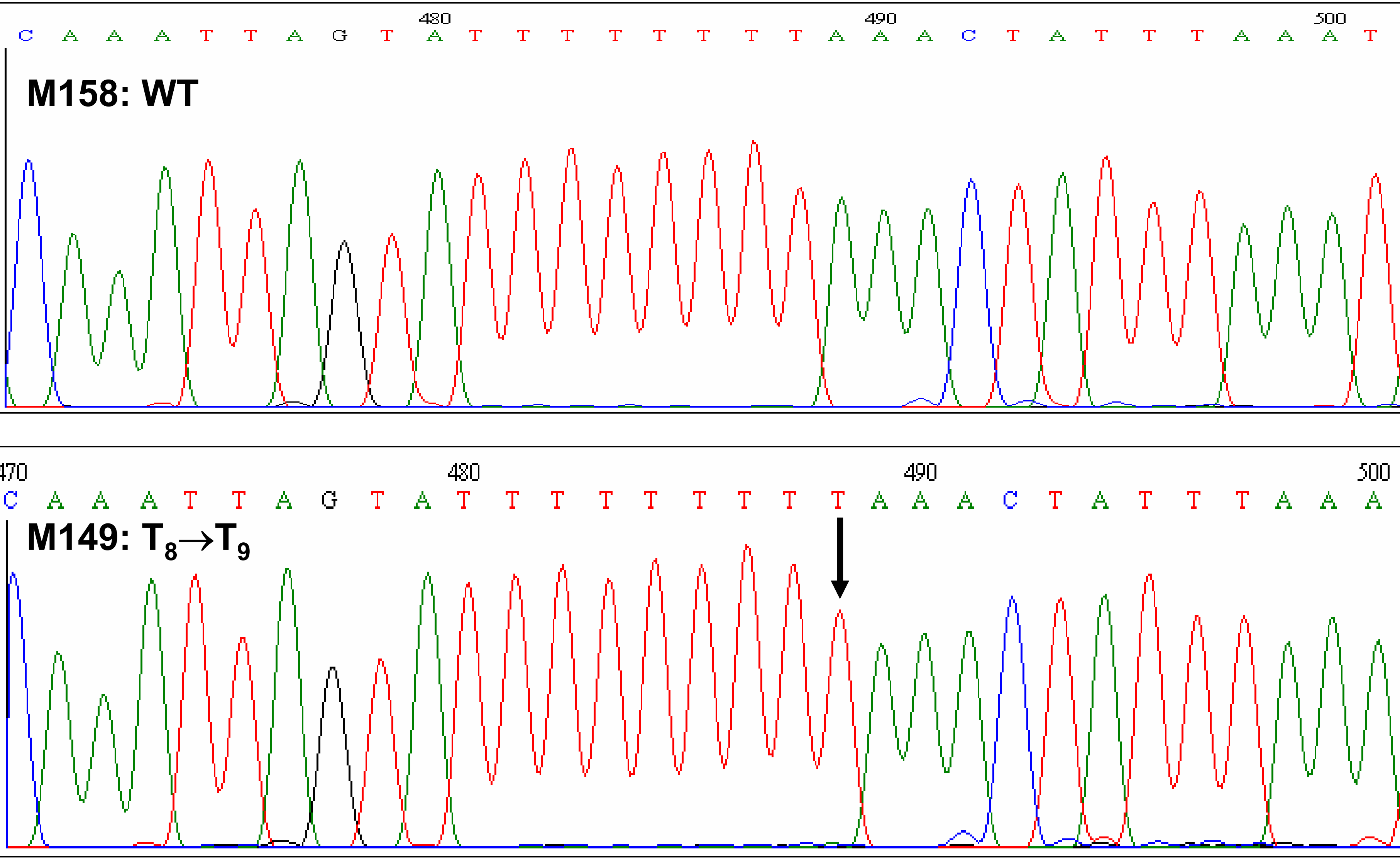

Position: 8,192

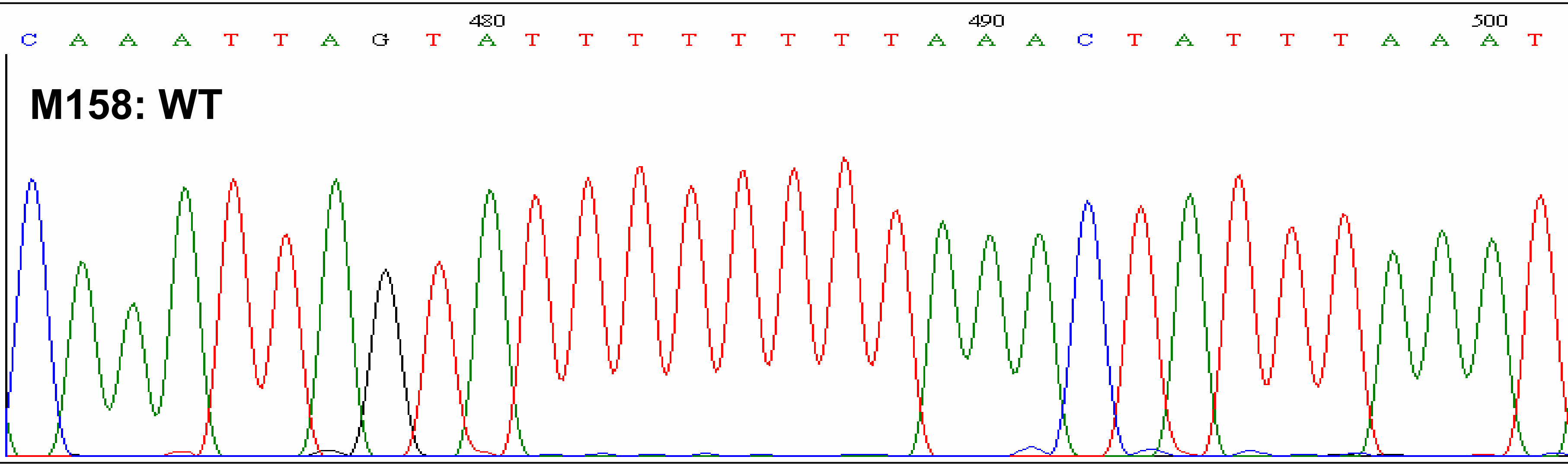

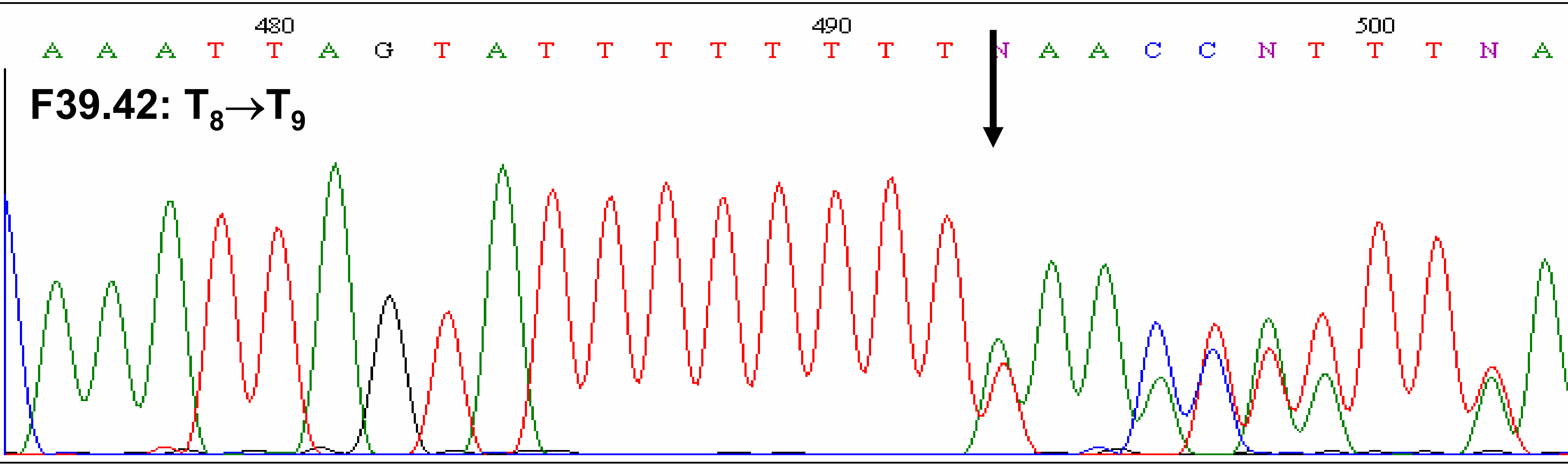

Position: 9,185

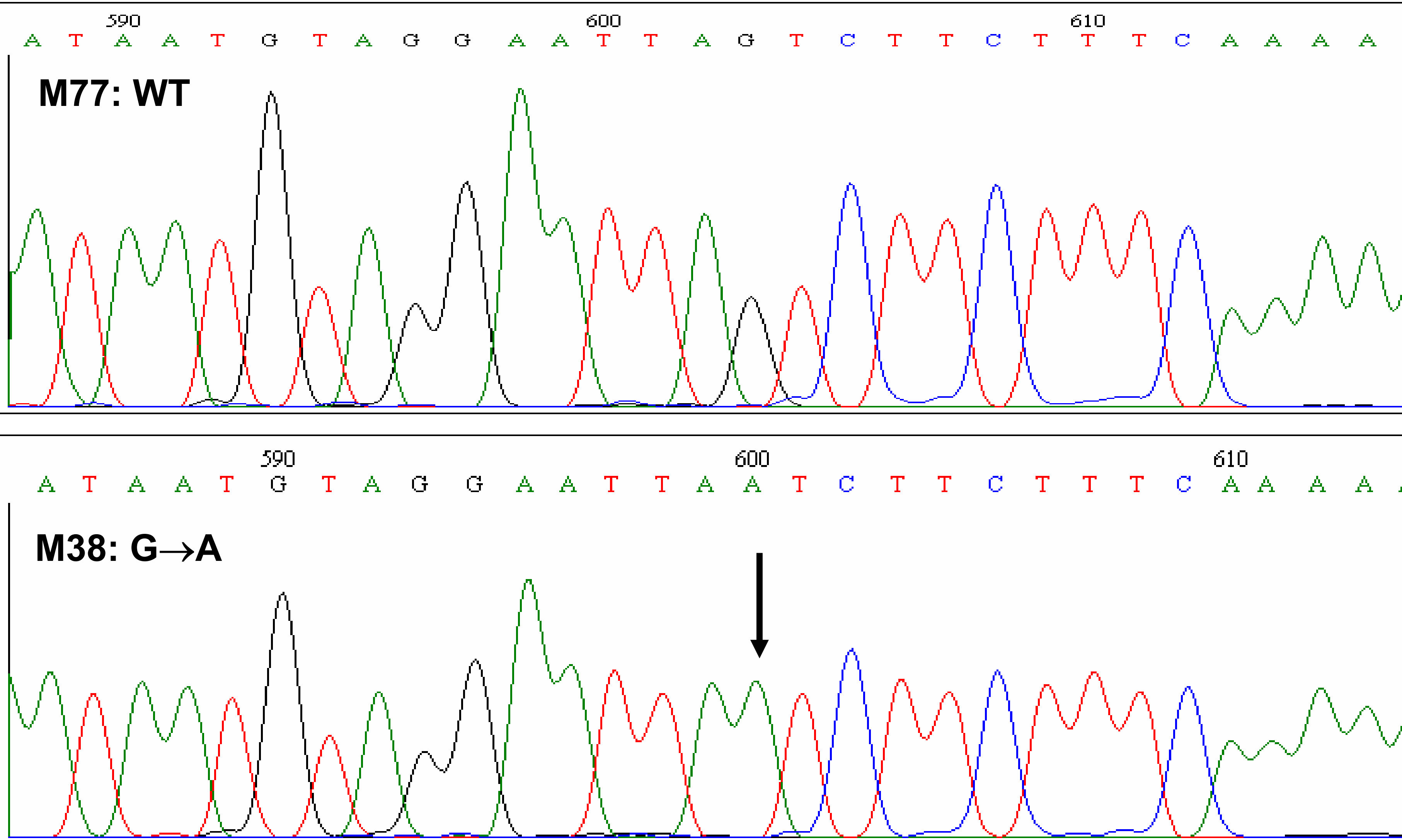

Position: 10,094

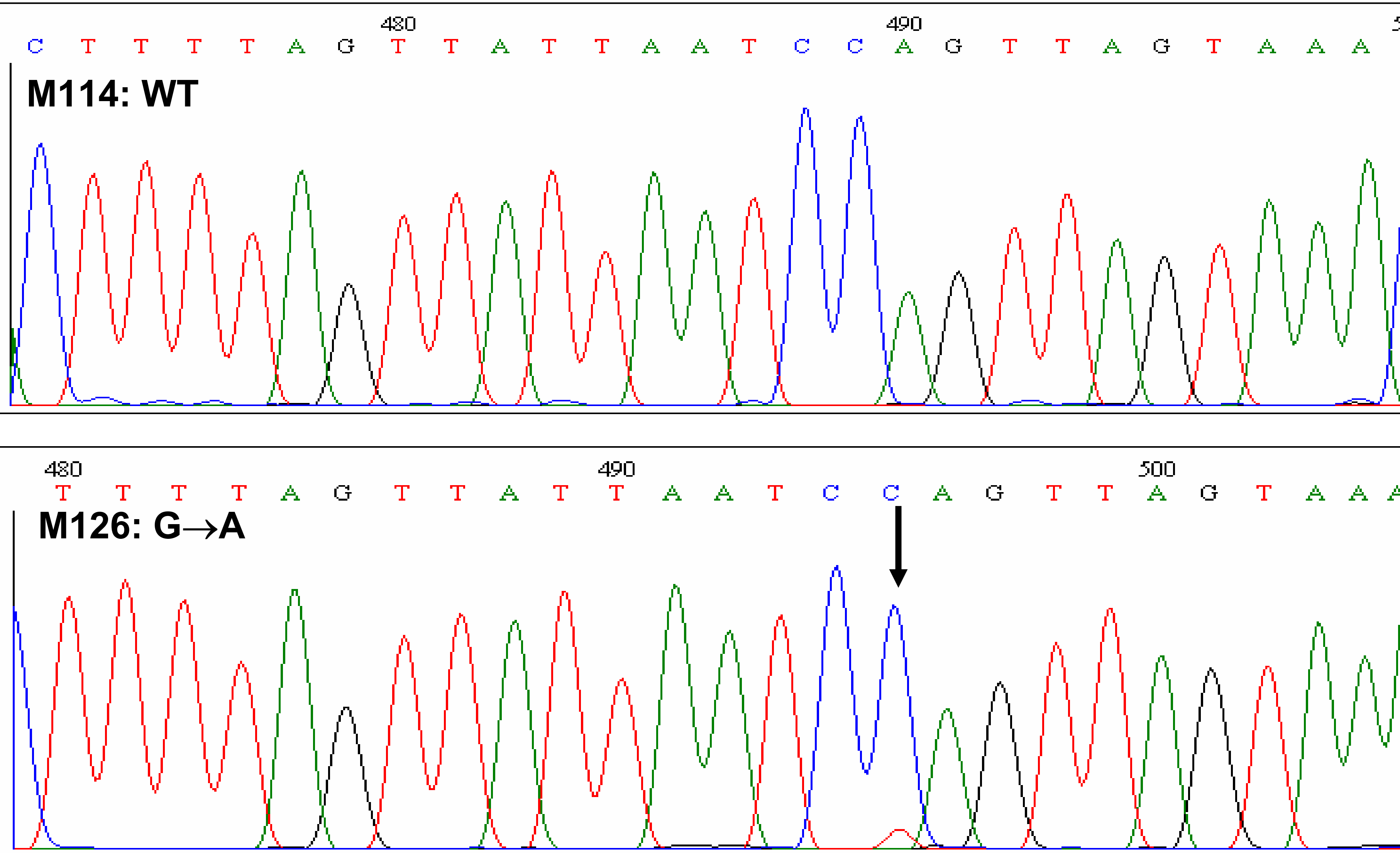

Mutation visible only on reverse sequence & observed in two independent PCRs/sequences

Position: 10,541

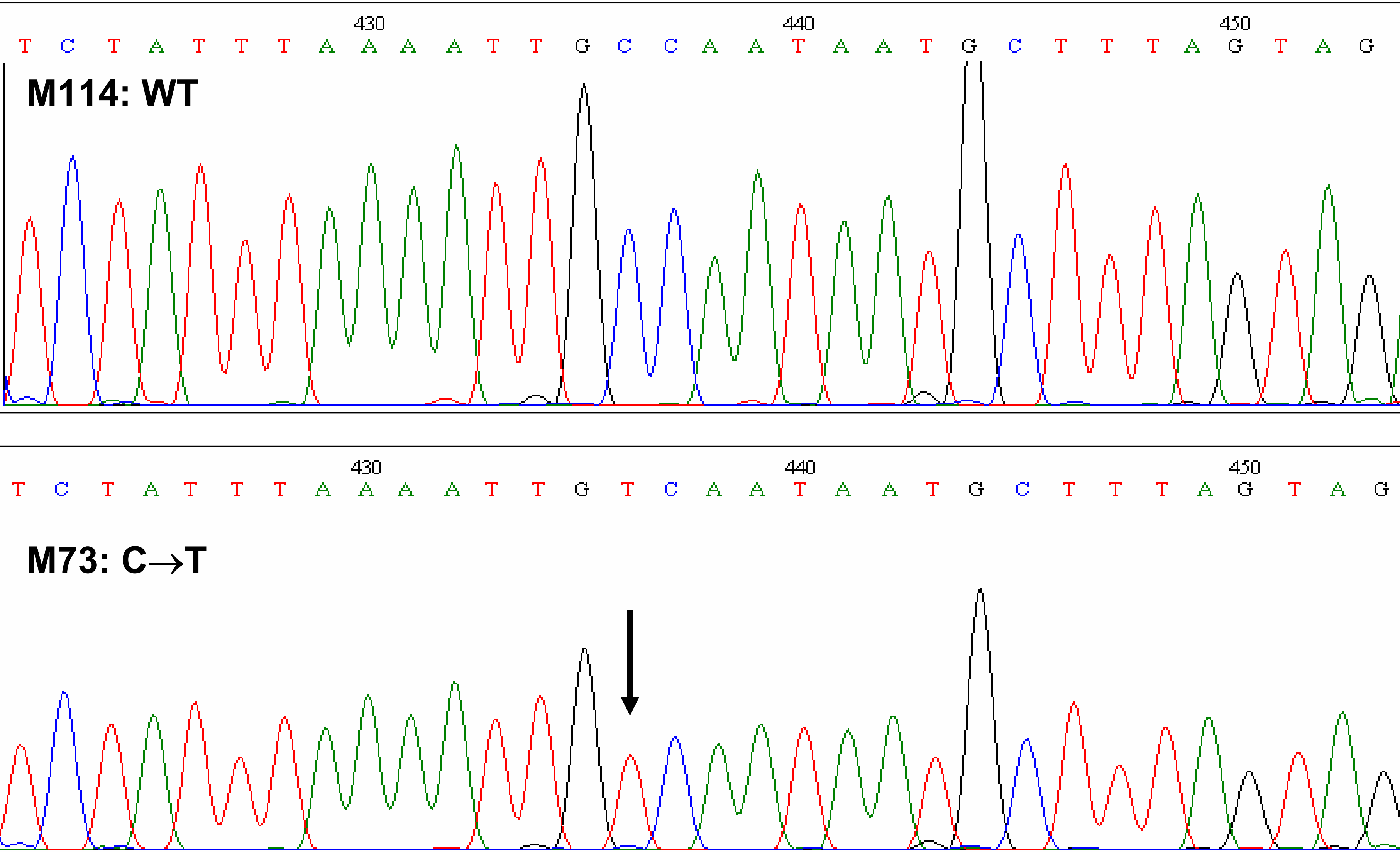

Position: 10,801 (NC)

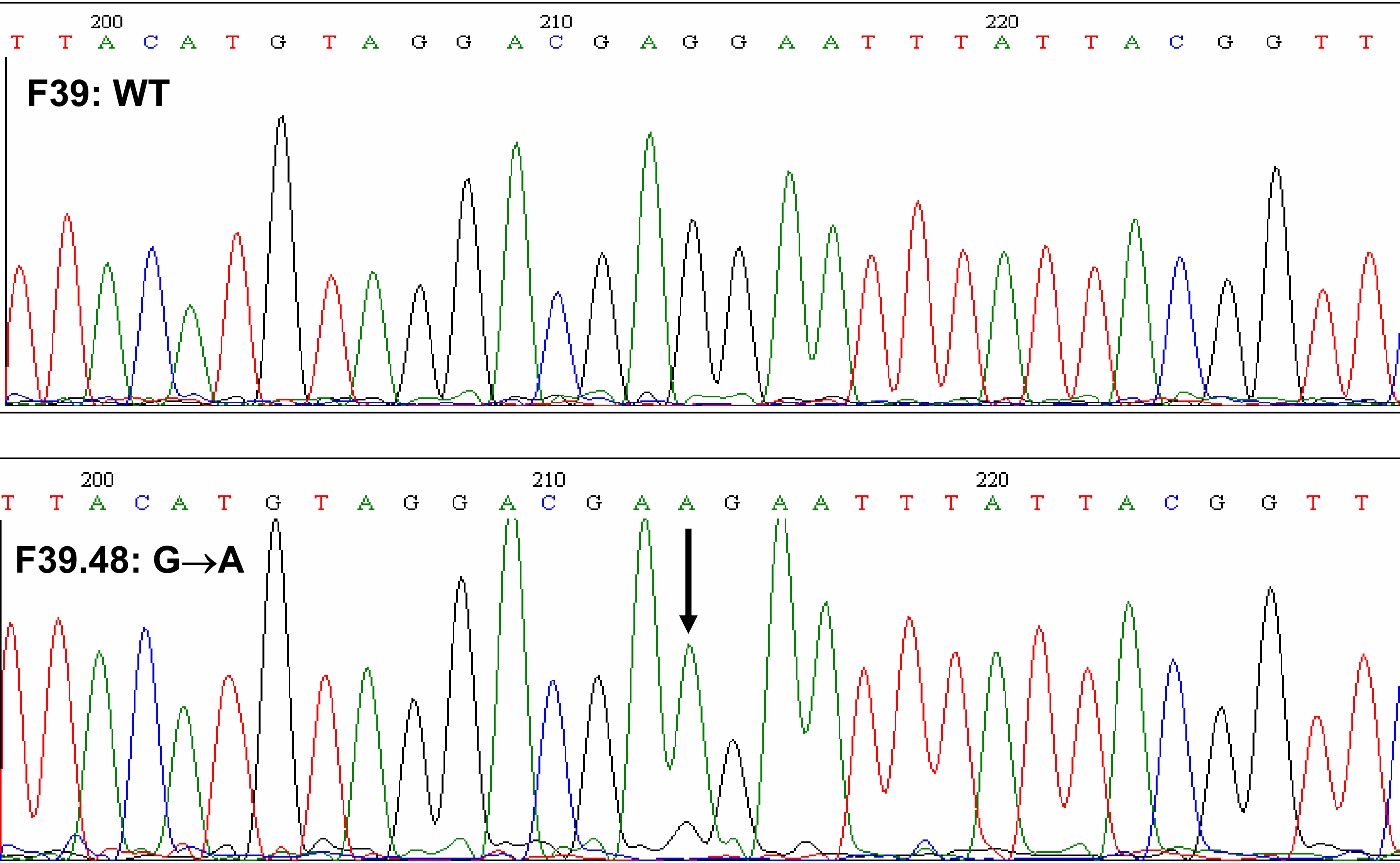

Position: 10,970

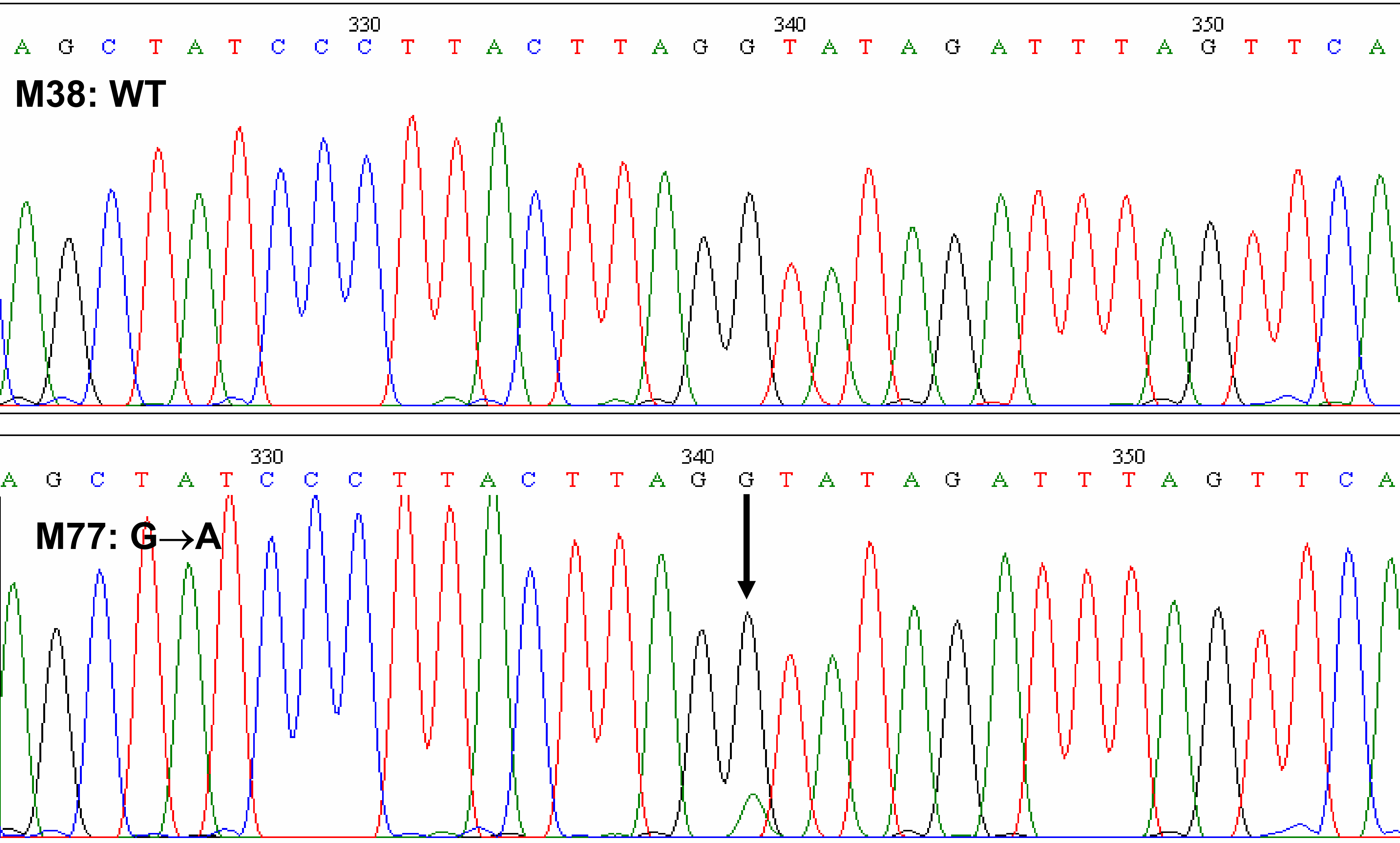

Position: 11,132

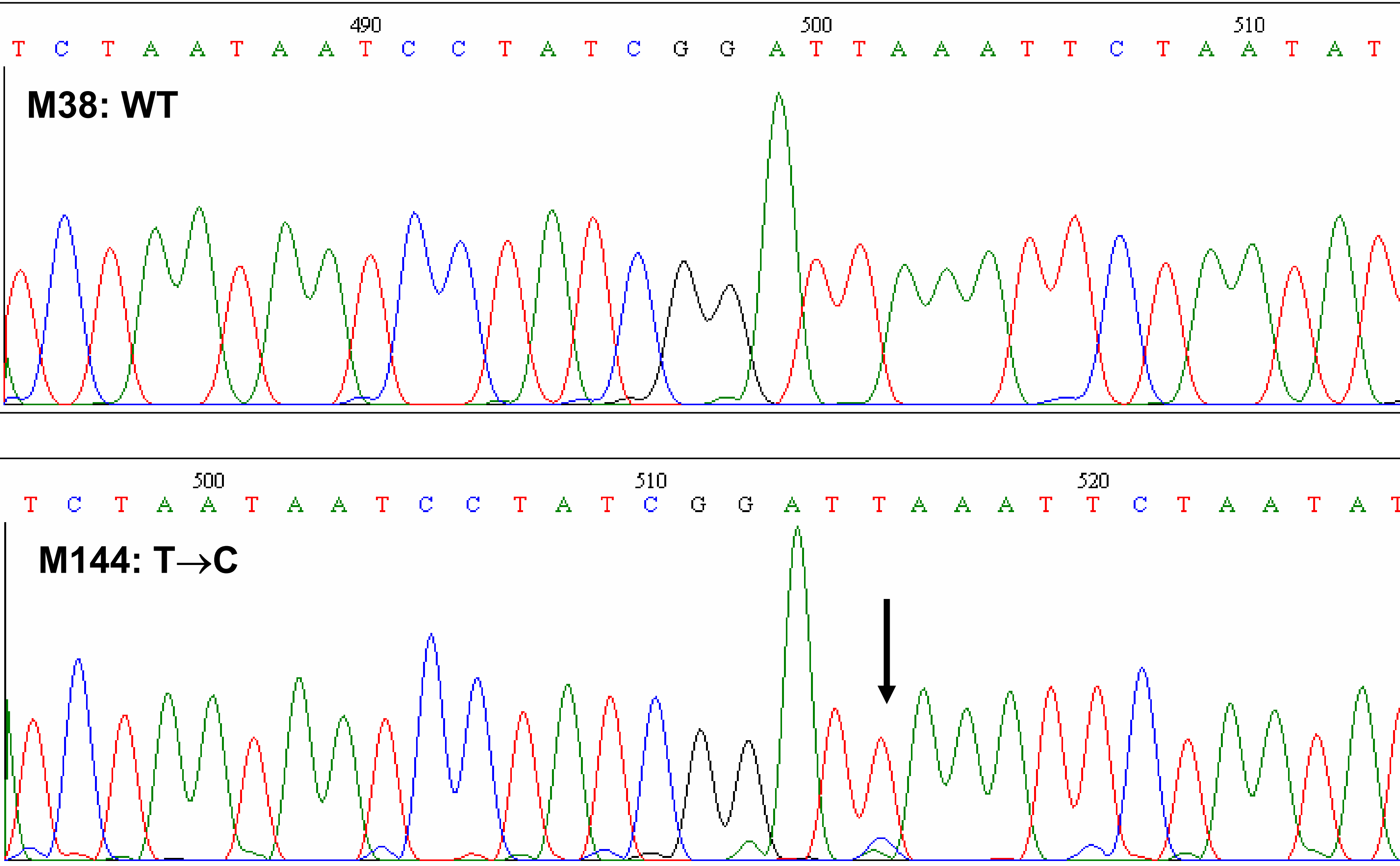

Position: 11,132

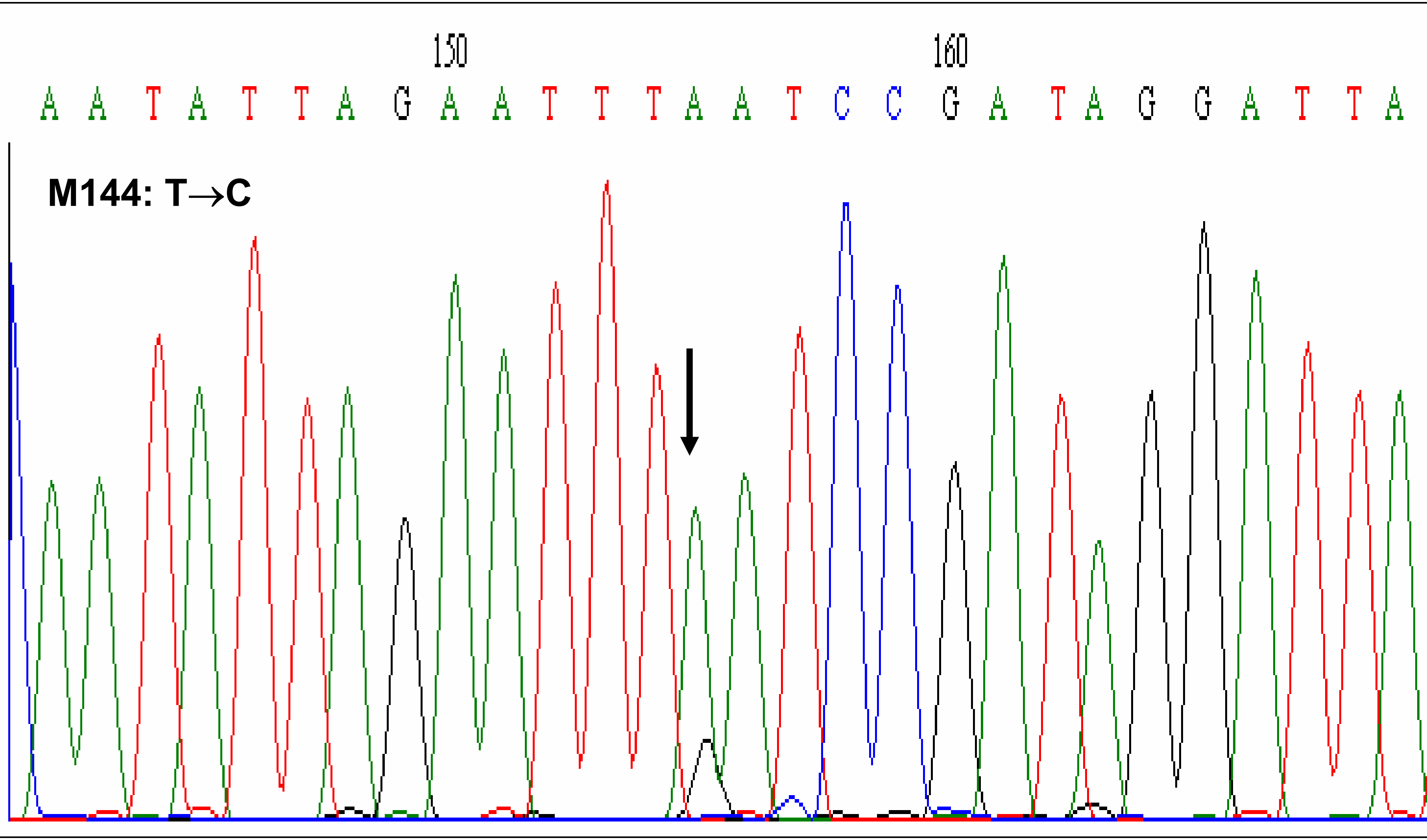

Same as previous mutation seen on reverse strand

Position: 11,542

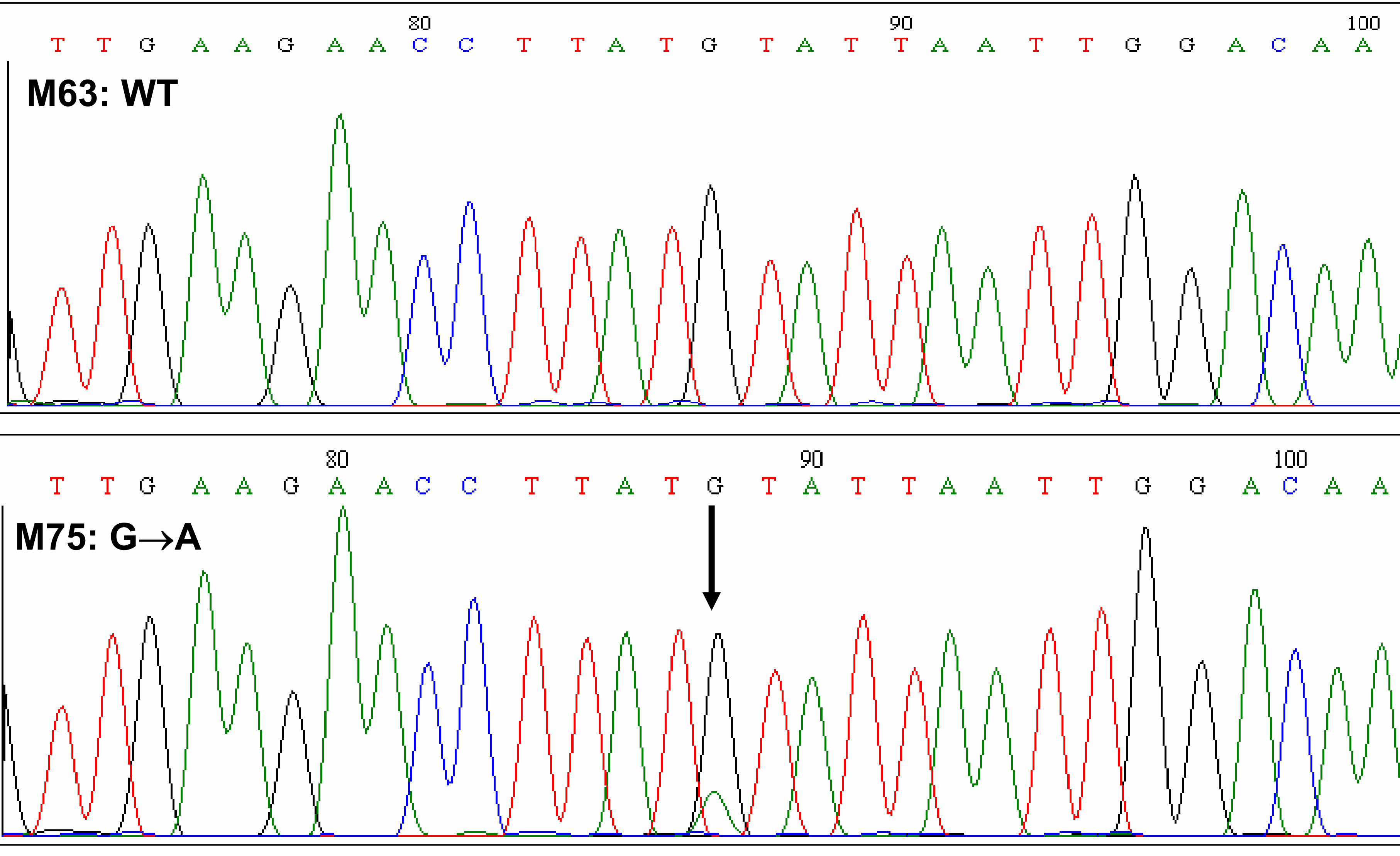

Position: 11,677

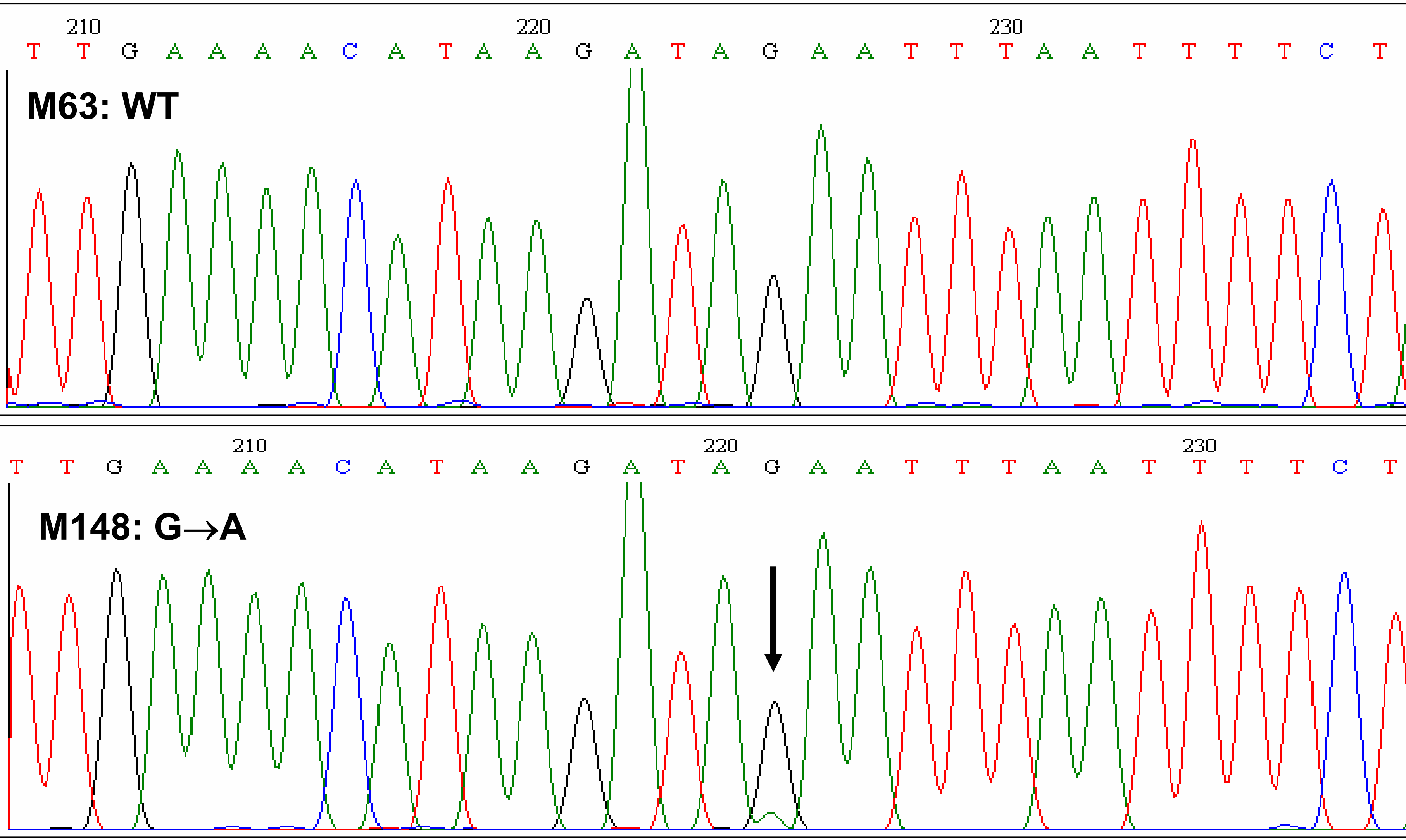

Found in two independent PCRs/sequences

Position: 11,708

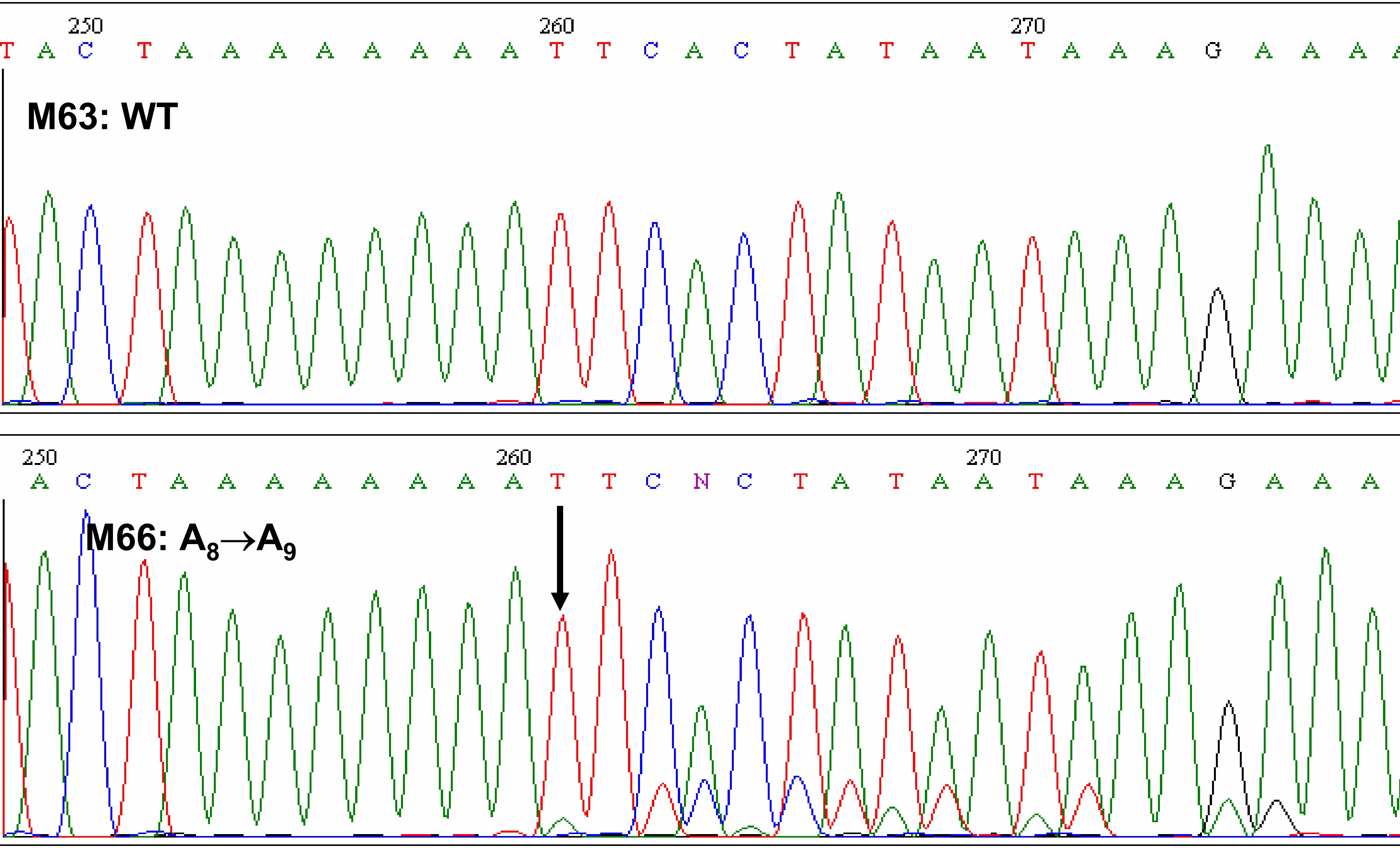

Position: 11,836

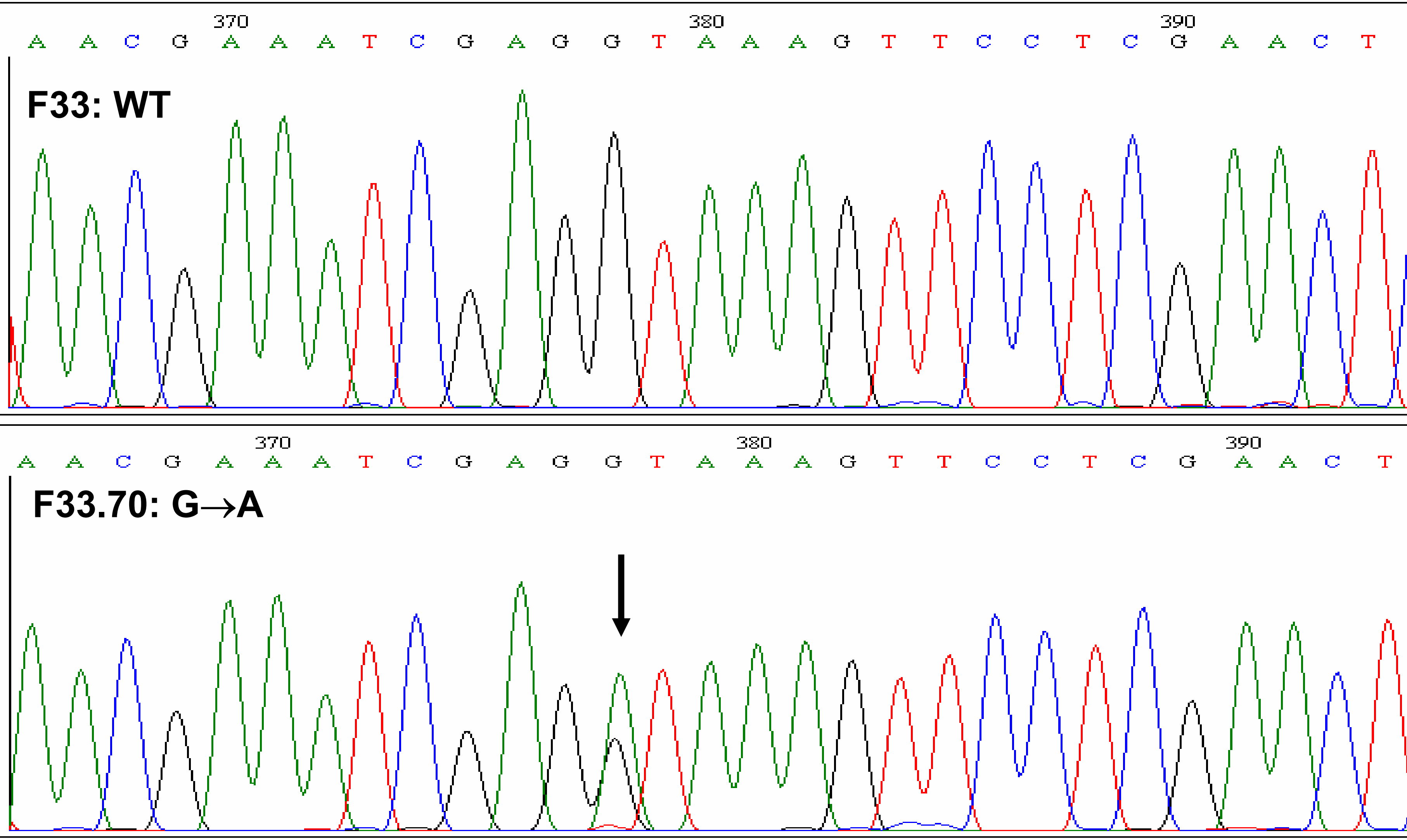

Position: 12,756

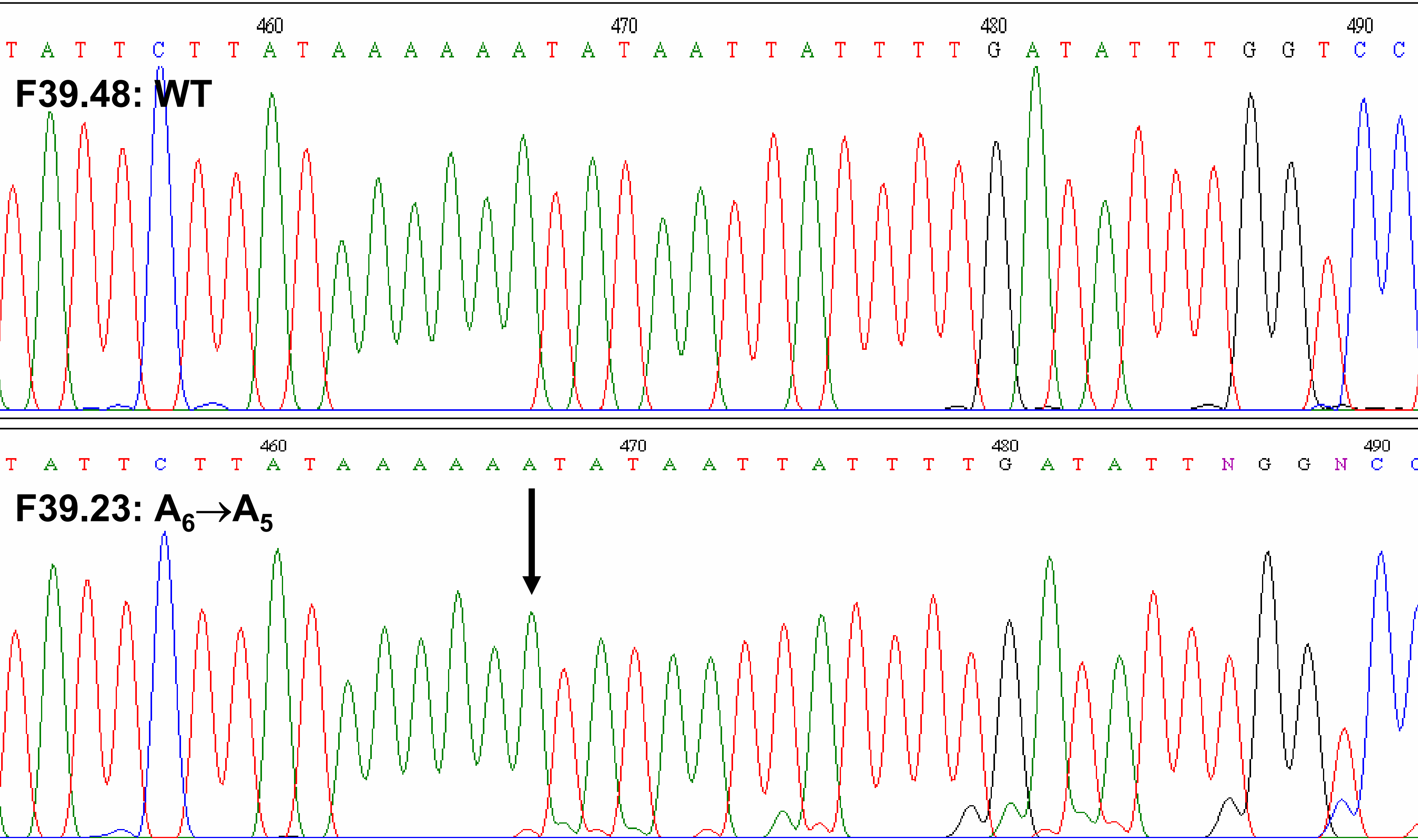

Position: 12,781

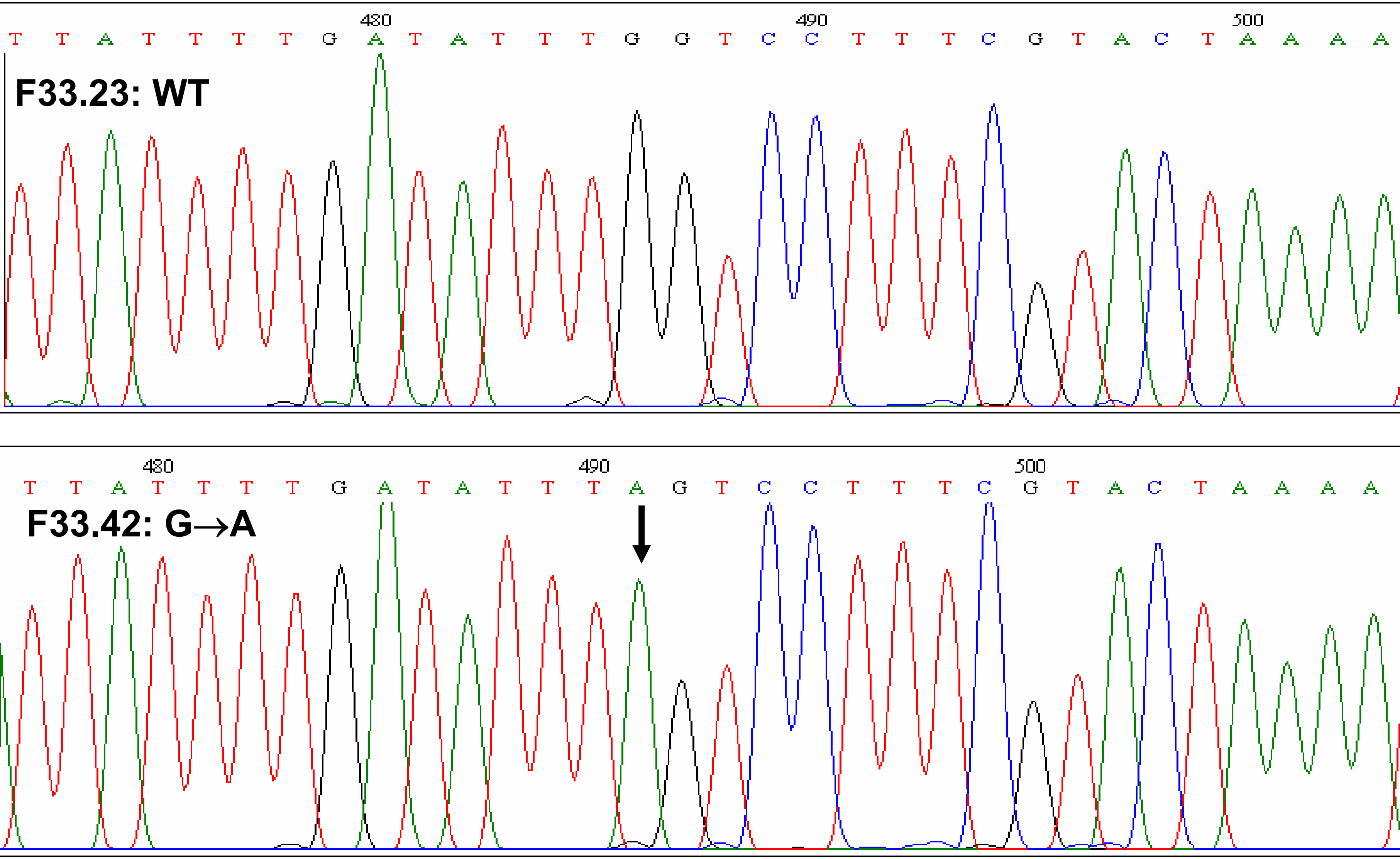

Position: 12,820

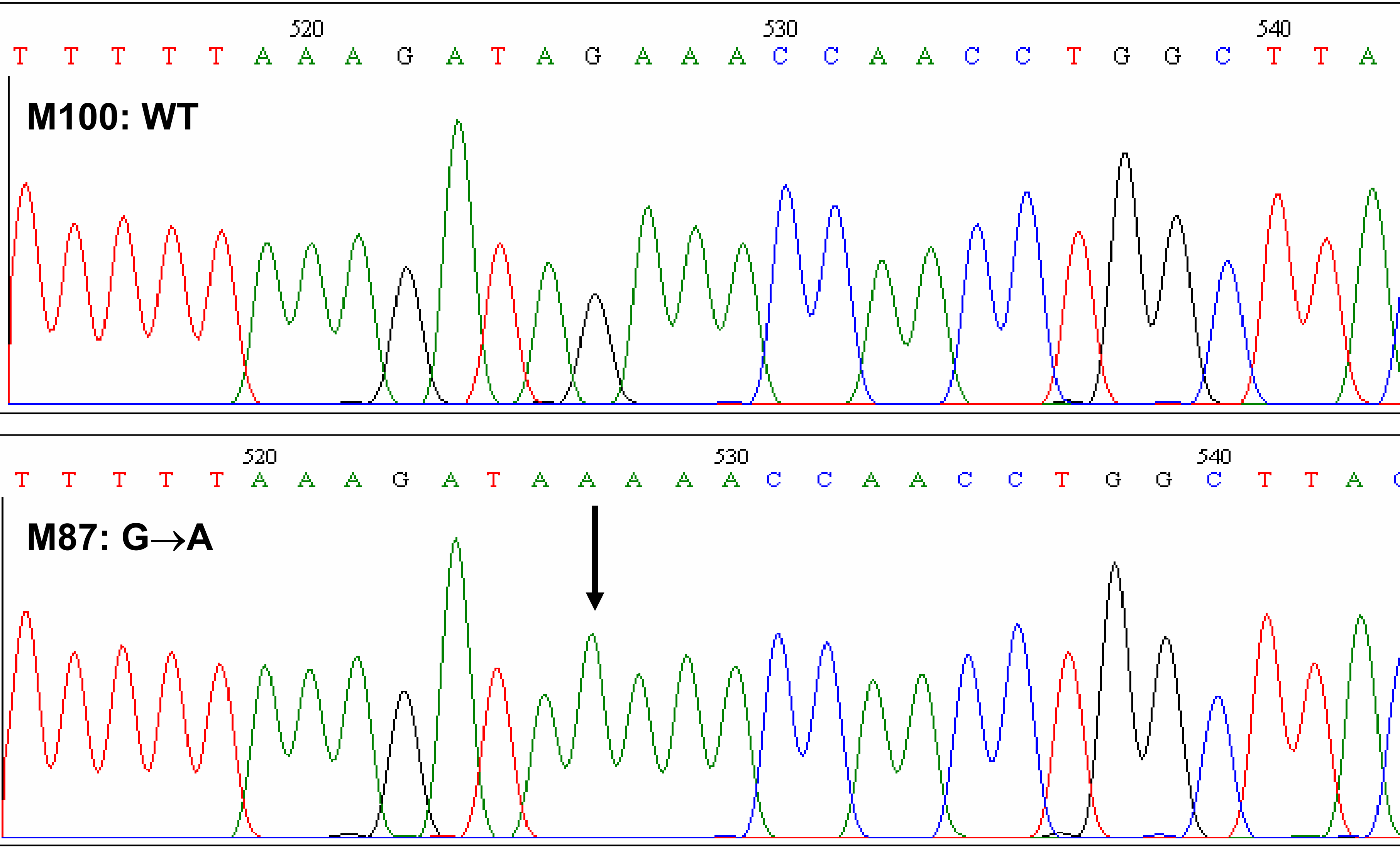

Position: 13,136

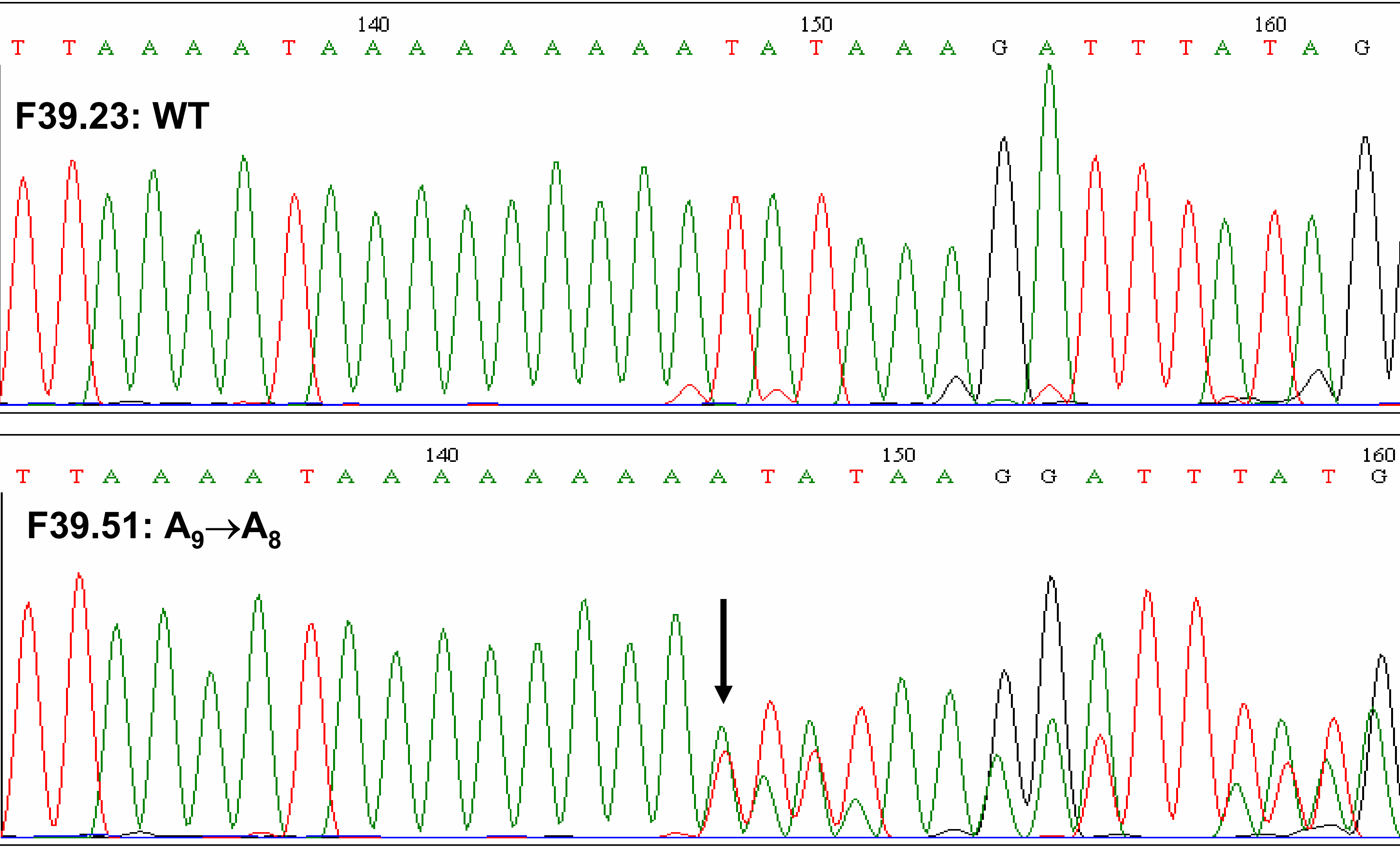

Position: 13,136

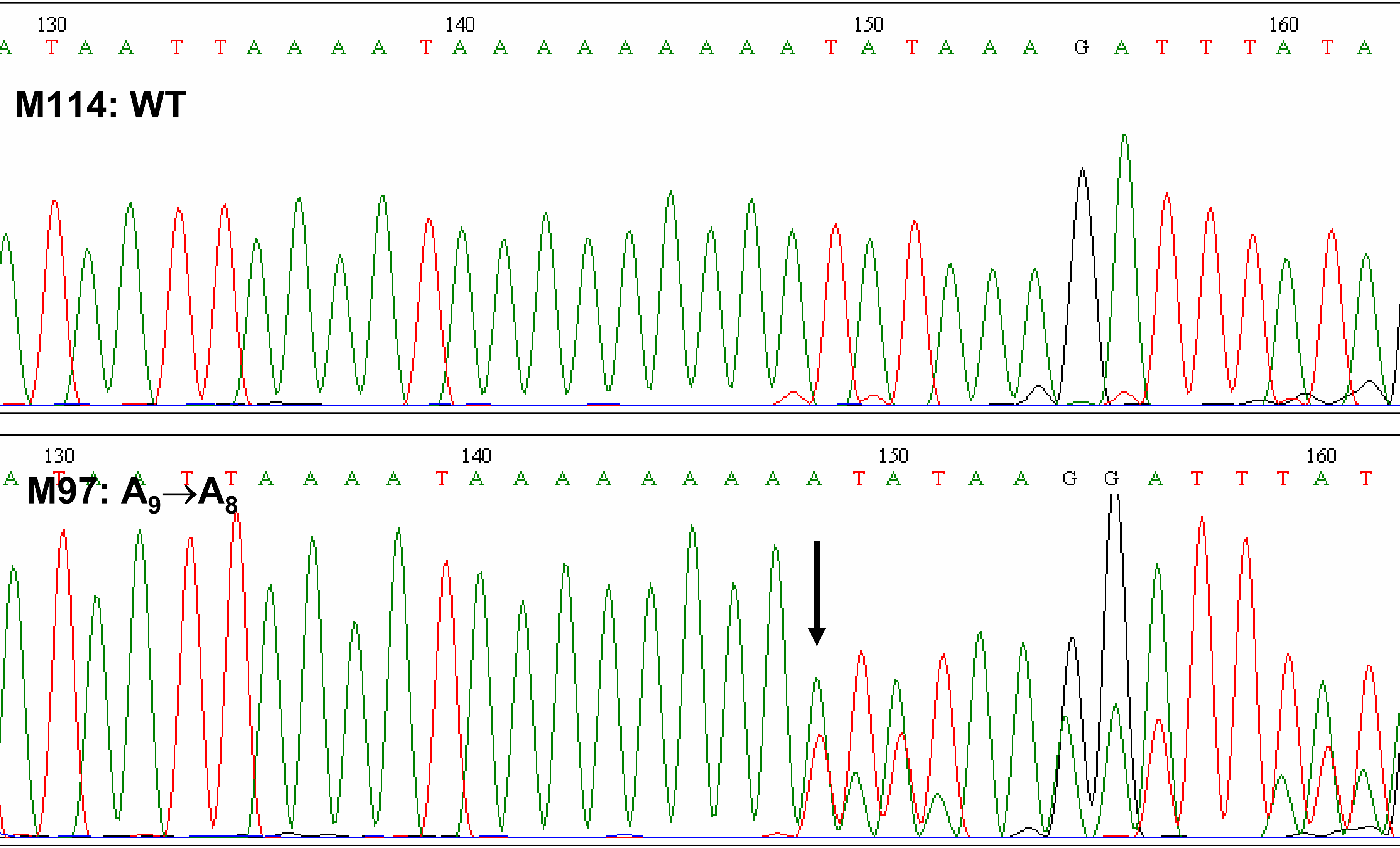

Position: 13,234

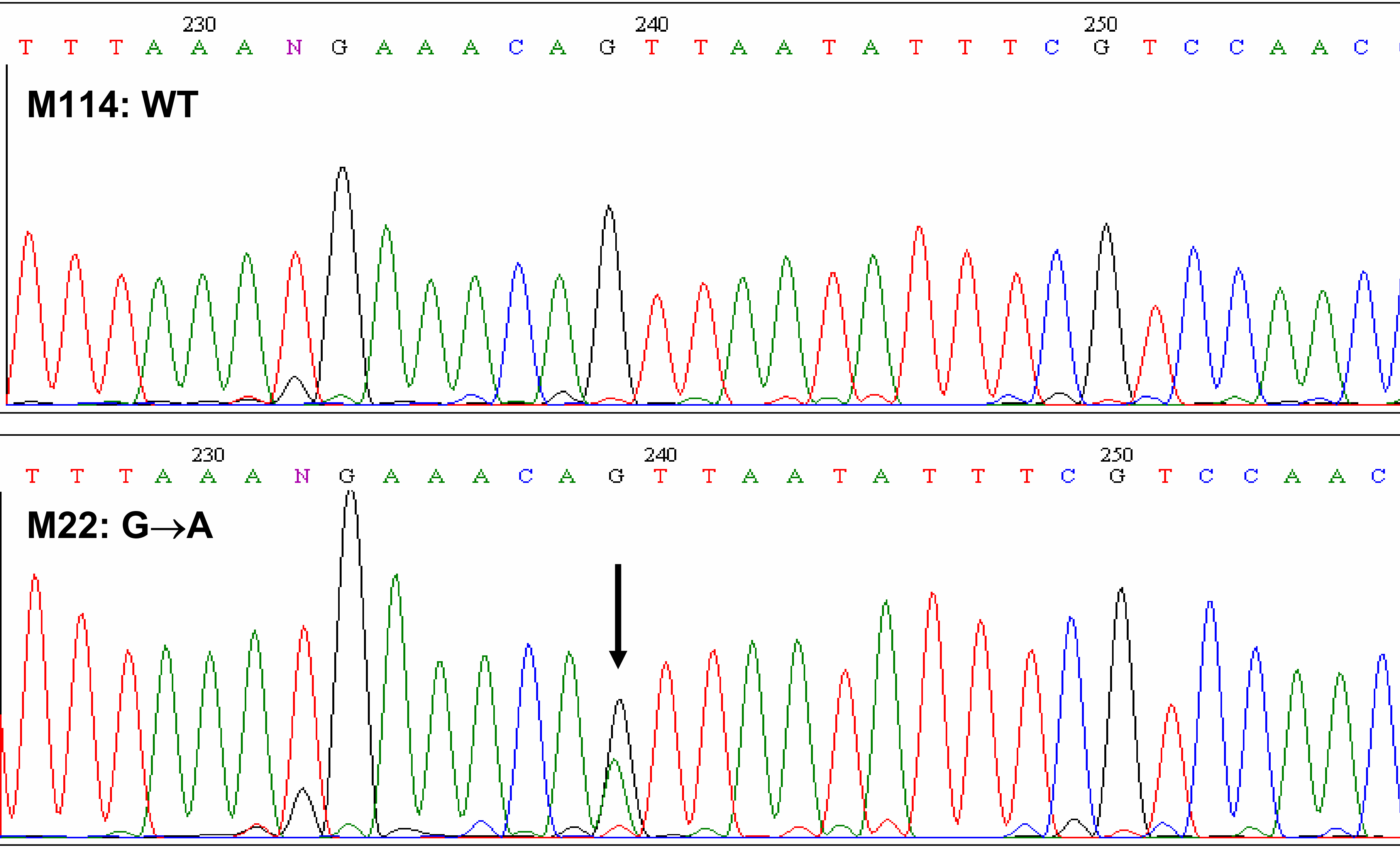

Position: 14,679

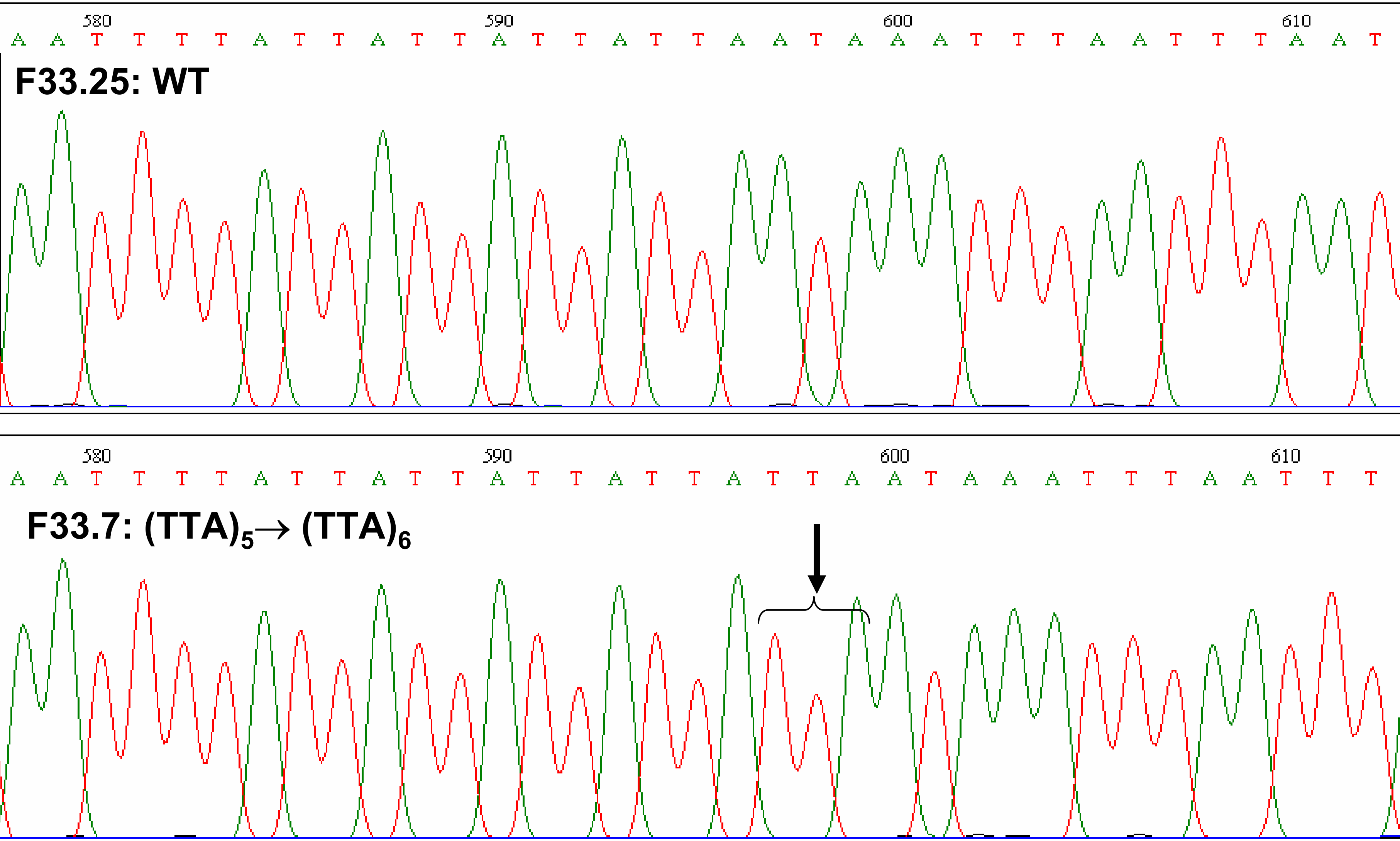

Supplement: Supplementary file 1 [file pbio.0060204.sd001.pdf]
