# Supplementary material for: Direct Estimation of the Mitochondrial DNA Mutation Rate in Drosophila melanogaster
Source: PLoS Biol. 2008 Aug 19;6(8):e204. doi: 10.1371/journal.pbio.0060204 (PMC2517619; doi:10.1371/journal.pbio.0060204)
Supplement: Supplementary file 2 [file pbio.0060204.st001.pdf]

Table S1. Regions of the *D. melanogaster* mt genome scanned by DHPLC and direct Sanger sequencing.

DHPLC			Sequencing		
Amplicon name	start 5'	end 3'	Amplicon name	start 5'	end 3'
NADH2a	12	761	339-1461	319	1479
COIa	1579	2279	1005-1930	981	1950
COIb	2288	2989	1852-2827	1827	2844
COII	3005	3706	2783-3647	2761	3664
ATPase 6-8	3650	4350	3598-4528	3570	4524
ATPase6	4424	5124	4463-5442	4440	5438
COIII	5140	5842	5314-6195	5288	6155
NADH3	5911	6660	6143-6969	6061	6924
NADH5a	6866	7614	6931-7740	6849	7701
NADH5b	7670	8371	7852-8896	7776	8858
NADH4	8549	9251	8780-9672	8704	9636
NADH4L bis	9563	10196	10626-11573	10565	11538
NADH6	10070	10620	11355-12329	11276	12294
Cytb	10595	11323	12312-13356	12236	13302
NADH1a	11421	12120	13321-14251	13246	14196
NADH1b	12263	13011	14266-14787	14149	14756
ribosome2	12968	13268			
ATregion1	14054	14736			
