# Supplementary material for: Direct Estimation of the Mitochondrial DNA Mutation Rate in Drosophila melanogaster
Source: PLoS Biol. 2008 Aug 19;6(8):e204. doi: 10.1371/journal.pbio.0060204 (PMC2517619; doi:10.1371/journal.pbio.0060204)
Supplement: Supplementary file 3 [file pbio.0060204.st002.pdf]

Table S2. Details of mutations in Florida lines detected by DHPLC, but not by direct sequencing, and *vice versa*.

		Detected by:		
Line	***Position***	***DHPLC***	***Sequencing***	***Frequency***
F33.122	7,382	Yes	No	0.18
F39.48	10,801	No	Yes	0.87
F33.70	11,836	Yes	No	0.54
F39.23	12,756	Yes	No	0.15
F39.51	13,136	Yes	No	0.45
F33.7	14,679	Yes	No	1.0
