# Supplementary material for: Direct Estimation of the Mitochondrial DNA Mutation Rate in Drosophila melanogaster
Source: PLoS Biol. 2008 Aug 19;6(8):e204. doi: 10.1371/journal.pbio.0060204 (PMC2517619; doi:10.1371/journal.pbio.0060204)
Supplement: Supplementary file 4 [file pbio.0060204.st003.pdf]

Table S3. Primers used for PCR amplification in mutation scanning by direct sequencing and DHPLC.

Direct sequencing approach

Locus Name	F-primer	R-primer
339-1461	GGTTAGGAGCTTGAATAGG	CGCGGTTATTGATTAAGTGGC
1005-1930	GGAGGACTACCCCCATTTTTAGG	GCTCCTAGAATTGAAGAAATTCC
1852-2827	CGGAGCTGGAACAGGATGAACTG	GTTGATCCAATAGTAGACAC
2783-3647	GGATTAGCTGGAATACCTCG	CCATAAAATAAACCTGGTCG
3593-4528	GAACAGTTCCCGCTTTAGGAG	GCAGTTAATCGGACAGCTAATGTCCC
4463-5442	GCTCACTTAGTCCCTCAAGG	CATGCAGCTGCTTCAAATCC
5314-6195	GCTCCATTTACTATTGCGGACTC	CATTAACAGTGATACGCCTC
6143-6969	GAAGCGATTGATTGCAGTTAGTTTCG	CTGGGGATTTAAATTGTGG
6946-7674	AGTATAATTCAACTTTCATC	TACAAAATGAATTTGAAATG
8780-9672	GAGCAACAGATGAATAAGC	GAGTATGTGAGGGAGCTTTAGG
10641-11573	CCAGCTCCAATTAATATCTC	GGTTCTTCGACTGGTCGAGCTCC
11355-12329	GCTAATCCTTTAGTTACACCTGC	GCTGGTTGATCTTCTAATTC
12312-13356	GAGCTACAGCTCGTAAACCTCC	GCCGCAGTATTTTGACTGTGC
13321-14251	CGTCCAACCATTCATTCCAGC	ATGTACACATCGCCCGTCGC
14226-14787	CCAGTACATCTACTATGTTACG	CCAAATTGGTGCCAGCAGTCGCGG

DHPLC approach

Locus Name	F-primer	R-primer
NADH2a	TGATAAAAAGGATTACCTTG	AATTTTCGTAAAGAAGTTTG
COIa	CGAGCTGAATTAGGACATCC	GAGAACCAAAAGTTTCCTTTTTTCC
COIb	TTTATGCTATATTAGCTATTG	TTCAGAATATCTATGTTCAG
COII	ATTAATTTCTAATATGGCAG	GGTATAAATCTATGATTAGC
ATPase 6-8	GGTTTATTTTATGGTCAATG	TGTTCTTGTAAAAATATATGG
ATPase6	TACACAACATATATTTGCTC	TTTGAAATGGATTAAATGAG
COIII	AGCTATTTTATTAGCTTCAG	AATTATAGGTAGAATTAATGC
NADH3	ATTGGATTATACCATGAATG	TTAAGAACTTTTTTAGGGTC
NADH5a	AGTATAATTCAACTTTCATC	TACAAAATGAATTTGAAATG
NADH5b	GAAAGAAGTAAAGCTACATC	CTGCTTATACTTTATATTTATATTC
NADH4	AATTAATATTCTTCGACTTC	TTGTTGATTTTAACTTTTTC

NADH4L bis	TGAAAATAATCATTACCATGAGTACG	TCATTAGAGGCTAAAGATGTTACG
NADH6	CAATTTTTGTATGTTTACTAAC	CATAATCCAAGTAATGATCC
Cytb1	ATTTTGGATCATTACTTGGATTATG	AAAATATCATTCTGGTTGAATATGG
NADH1a	TAATTTAAGAAAATTCCGAG	TTATTTTATTTCCTATAGCTTTAG
NADH1b	AGCATAATTAGAATTAGACG	GATTAAAAAATTAAGTTACTTTAGG
ribosome 2	AAAAATTACGCTGTTATCCCTAAAG	AGGCTGGAATGAATGGTTGG
ATregion1	CTAACTCAATTTATATTGATTTGC	CGCGGTTATACCATTAATAC
